# Symptom‐led staging for semantic and non‐fluent/agrammatic variants of primary progressive aphasia

**DOI:** 10.1002/alz.13415

**Published:** 2023-08-07

**Authors:** Chris J. D. Hardy, Cathleen Taylor‐Rubin, Beatrice Taylor, Emma Harding, Aida Suarez Gonzalez, Jessica Jiang, Laura Thompson, Rachel Kingma, Anthipa Chokesuwattanaskul, Ffion Walker, Suzie Barker, Emilie Brotherhood, Claire Waddington, Olivia Wood, Nikki Zimmermann, Nuriye Kupeli, Keir X. X. Yong, Paul M. Camic, Joshua Stott, Charles R. Marshall, Neil P. Oxtoby, Jonathan D. Rohrer, Anna Volkmer, Sebastian J. Crutch, Jason D. Warren

**Affiliations:** ^1^ Dementia Research Centre UCL Queen Square Institute of Neurology UCL London UK; ^2^ Uniting War Memorial Hospital Sydney Australia; ^3^ Faculty of Medicine Health and Human Sciences Macquarie University Sydney Australia; ^4^ Centre for Medical Image Computing Department of Computer Science UCL London UK; ^5^ Division of Neurology Department of Internal Medicine King Chulalongkorn Memorial Hospital Bangkok Thailand; ^6^ Cognitive Clinical and Computational Neuroscience Research Unit Faculty of Medicine Chulalongkorn University Bangkok Thailand; ^7^ HealthAbility Melbourne Australia; ^8^ Marie Curie Palliative Care Research Department Division of Psychiatry UCL London UK; ^9^ ADAPT Lab Research Department of Clinical Educational and Health Psychology UCL London UK; ^10^ Preventive Neurology Unit Queen Mary University of London London UK; ^11^ Psychology and Language Sciences (PALS) UCL London UK

**Keywords:** frontotemporal dementia, primary progressive aphasia, primary progressive non‐fluent/agrammatic aphasia, progression planning aid, semantic dementia, staging

## Abstract

**INTRODUCTION:**

Here we set out to create a symptom‐led staging system for the canonical semantic and non‐fluent/agrammatic variants of primary progressive aphasia (PPA), which present unique diagnostic and management challenges not well captured by functional scales developed for Alzheimer's disease and other dementias.

**METHODS:**

An international PPA caregiver cohort was surveyed on symptom development under six provisional clinical stages and feedback was analyzed using a mixed‐methods sequential explanatory design.

**RESULTS:**

Both PPA syndromes were characterized by initial communication dysfunction and non‐verbal behavioral changes, with increasing syndromic convergence and functional dependency at later stages. Milestone symptoms were distilled to create a prototypical progression and severity scale of functional impairment: the PPA Progression Planning Aid (“PPA‐Squared”).

**DISCUSSION:**

This work introduces a symptom‐led staging scheme and functional scale for semantic and non‐fluent/agrammatic variants of PPA. Our findings have implications for diagnostic and care pathway guidelines, trial design, and personalized prognosis and treatment for PPA.

**Highlights:**

- We introduce new symptom‐led perspectives on primary progressive aphasia (PPA).
- The focus is on non‐fluent/agrammatic (nfvPPA) and semantic (svPPA) variants.
- Foregrounding of early and non‐verbal features of PPA and clinical trajectories is featured.
- We introduce a symptom‐led staging scheme for PPA.
- We propose a prototype for a functional impairment scale, the PPA Progression Planning Aid.

## 1 INTRODUCTION

Primary progressive aphasias (PPA) are language‐led dementias that pose challenges for diagnosis and management.^1^, ^2^, ^3^, ^4^, ^5^, ^6^, ^7^, ^8^, ^9^, ^10^ Information on the evolving daily‐life impact of PPA is limited:^9^ there is no clear “roadmap” for the development of deficits/disability and care planning for patients and families.^4^, ^5^ While the value of clinical staging in neurodegenerative diseases is widely acknowledged,^9^, ^11^, ^12^, ^13^, ^14^ assessing disease burden objectively in dementia is problematic, the pathophysiological milestones of disease progression are often unknown, and there is wide individual variation in phenotypic expression. All these challenges are amplified in PPA, reflecting the complexity of language functions, a comparative lack of reliable in vivo progression biomarkers and marked phenotypic heterogeneity.^2^, ^3^, ^15^, ^16^, ^17^

The canonical syndromes of PPA comprise the non‐fluent/agrammatic variant (nfvPPA), led by impaired speech production and/or agrammatism; the semantic variant (svPPA), led by breakdown of vocabulary and semantic memory for non‐verbal objects and concepts; and the logopenic variant (lvPPA), presenting with anomia and reduced verbal short‐term memory.^15^, ^18^ This formulation masks considerable variability, and excludes a substantial minority of cases not meeting criteria for a canonical syndrome, even early in the illness.^2^, ^3^, ^6^, ^19^ Clinical experience suggests that patients with these conditions transition through differentiable stages of impairment and functional disability:^20^ a “stage” here constituting a constellation of problems, developing as part of a sequence that is broadly similar among patients with each subtype. Early, there is often loss of facility with structured verbal exchanges and subtle changes in social behavior, while late‐stage disease tends to exhibit motor and other physical impairments, often accompanied by profound behavioral changes.^6^, ^8^, ^10^ Clinical experience further suggests that the sequence of impairments follows a trajectory that differs among syndromes.^10^, ^21^

Existing staging instruments rest primarily on concepts of disease impact formulated by clinicians, rather than the lived experience of patients and caregivers. Moreover, concepts of clinical progression underpinning these instruments have often been informed by experience with the most common dementia, Alzheimer's disease (AD). Standard instruments^11^, ^22^ do not assess language and communication functions sufficiently for staging PPA. Clinical severity rating scales more relevant to PPA—notably the Frontotemporal Dementia Rating Scale (FRS),^12^ Clinical Dementia Rating + National Alzheimer's Disease Coordinating Center Frontotemporal Lobar Degeneration scale,^23^, ^24^ and Progressive Aphasia Severity Scale (PASS)^25^—do not cover the full gamut of symptoms associated with these syndromes. The FRS, for example, lacks granularity for assessing communication functions while the PASS does not include non‐language symptoms, which may dictate the functional impact of evolving PPA.^6^, ^7^, ^10^, ^26^, ^27^, ^28^ Whereas lvPPA is most often underpinned by AD pathology and shows extensive convergence with other AD variants,^28^, ^29^, ^30^ nfvPPA and svPPA are usually associated with non‐AD pathologies in the frontotemporal dementia spectrum and have phenotypic features that are not encompassed by staging schemes oriented to AD. Accordingly, nfvPPA and svPPA are our focus in this study.

**FIGURE 1 alz13415-fig-0001:**
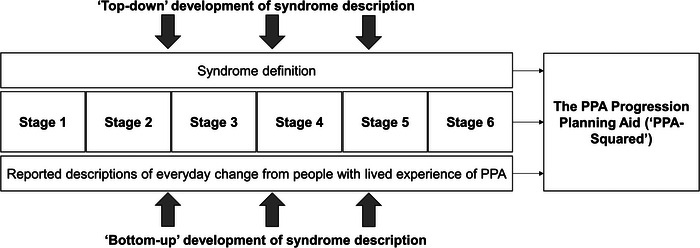
Development of the Primary Progressive Aphasia Progression Planning Aid (PPA−Squared). The figure illustrates the different sources of information incorporated in PPA‐Squared. Data were synthesized from “top‐down” sources to characterize what was known from a clinician/researcher perspective (i.e., clinician‐led interpretation of patient records, histories, and neuropsychological test scores) and from “bottom‐up” sources to capture crucial information from those with lived experience of the conditions (i.e., patient/caregiver‐derived symptoms collected on surveys, and organized, prioritized, and amended to reflect their lived experience of disease progression). We present two levels of illness description that clinicians and people with lived experience of PPA may find useful: the initial six stage ordering with a granular overview of specific symptoms (see Figures 2 and 3); and the PPA‐Squared (Table 3), a distillation of the survey data to create a clinically applicable staging scheme for clinician scoring of illness progression and severity, under the same broad functional domains presented in the figures.

Here we set out to generate a databank with granular detail about symptom evolution in these PPA syndromes; and to derive from this a clinical staging scheme and a prototypical, symptom‐led, functional impairment rating scale, the PPA Progression Planning Aid (“PPA‐Squared”). We envisaged this enterprise as synthesizing “top‐down” expertise of researchers and clinicians and “bottom‐up” perspectives of those with lived experience of PPA (Figure 1). Data were collected from a large, international cohort of English‐speaking patients with PPA and primary caregivers. Following previous approaches,^31^, ^32^, ^33^ caregivers completed a detailed survey on symptom development in PPA, tapping their lived experience. Acknowledging the important roles of both quantitative and qualitative feedback, we adopted a mixed‐methods sequential explanatory design.^34^ Our guiding objectives were to obtain from the surveys a detailed picture of the evolution of svPPA and nfvPPA, based on lived experience; to collate survey data into succinct descriptions of different “stages” of illness evolution; to capture qualitatively caregivers’ impressions of illness impact and trajectory and the value of the staging exercise; and to distill the survey data into a progression and severity rating scale of daily life functional impairment, highlighting clinical milestones of illness evolution.

## 2 METHODS

### 2.1 Exploratory survey

An initial putative list and ordering of PPA symptoms was proposed by two of the authors (C.J.D.H. and J.D.W.), based on clinical observations in the Dementia Research Centre PPA cohort, detailed review of case notes for patients with PPA attending the Specialist Cognitive Disorders Clinic at the National Hospital for Neurology and Neurosurgery, University College London, and a narrative review of the published literature on PPA (summarized in the Introduction). Symptoms covered domains of verbal communication (A) and non‐verbal functioning (non‐verbal thinking and personality, B1; personal care and well‐being, B2). Using an online survey hosted on the Opinio platform, qualitative feedback on the symptoms was gathered from 84 caregivers for people with a canonical syndrome of PPA who were registered with the United Kingdom national PPA Support Group.^35^ Data were collected between October 2018 and January 2019, and respondents comprised 41 caregivers of people with svPPA, and 43 with nfvPPA; all had had longstanding personal contact with the patients whose illness they described. Based on this survey, we expanded the list of symptoms and created a provisional six‐stage framework for ordering symptoms of functional impairment in svPPA and nfvPPA separately, ranging from stage 1 (least severe) to 6 (most severe). To aid caregivers in responding, we added descriptions of the daily life impacts for each stage, broadly based on the Reisberg Global Deterioration Scale^11^ (Table S1 in supporting information) as well as descriptors used previously in stages for another rare dementia, posterior cortical atrophy;^36^ see Table S2 and Table S3 in supporting information.

**RESEARCH IN CONTEXT**

1. **Systematic review**: We conducted a narrative literature review using traditional (e.g., PubMed) sources and meeting abstracts. While staging instruments are available for primary progressive aphasia (PPA), these rest primarily on concepts of disease severity and evolution formulated by clinicians, rather than the lived experience of patients and caregivers.
2. **Interpretation**: Our findings argue for a fresh consensus on diagnostic and management guidelines for semantic (svPPA) and non‐fluent/agrammatic (nfvPPA) variants, to take account of important emerging themes in the clinical phenotyping of these patients, and highlight the importance of learning from people with lived experience of these conditions, in conjunction with clinical and research‐derived observations.
3. **Future directions**: We propose a symptom‐led staging scheme and functional scale for svPPA and nfvPPA: the PPA Progression Planning Aid (“PPA‐Squared”). We hope this work will aid clinical care and motivate multidimensional, international collaborations to take the essential (and challenging) next steps toward validation.

### 2.2 Consolidation of the provisional stages

The provisional stages for each PPA syndrome were next entered into another online, mixed‐methods “consolidation” survey, designed[Fig alz13415-fig-0001] to allow us to refine the provisional staging framework. This second survey was refined for comprehensibility and presentation based on feedback from the exploratory survey and published guidelines for online research survey design,^37^ and was again hosted on the Opinio platform. This voluntary survey was distributed via e‐mail to caregivers comprising members of the UK PPA Support Group and PPA support groups in Melbourne and Sydney, Australia. Both current and bereaved caregivers were surveyed, to allow us to include information about late‐stage disease, and data were collected between February 2020 and April 2020 for UK PPA Support Group respondents and between January 2021 and May 2021 for Australian support group respondents. All caregivers had again had longstanding personal contact with the patients whose illness they described.

At the top of the survey, caregiver respondents first identified the major syndromic diagnosis for which they were filling out the survey and provided information about their relationship to the patient, and the patient's age currently, at symptom onset, when first assessed medically, and when diagnosed. The symptom labels presented to respondents in the consolidation survey are given in full in Tables S2 and S3. Customized symptom lists were presented under each stage according to the syndromic PPA diagnosis with which the respondent self‐identified at the top of the survey; this was to ensure respondent caregivers were able to focus on symptoms most relevant to “their” syndrome, while keeping their task manageable. A given PPA stage will be defined by a particular conjunction of symptoms; however, there was no prior “ground truth” to determine the correct conjunction for each stage. For each symptom, survey respondents were therefore asked to indicate whether, based on proximity to other symptoms and the overarching stage description (Table S1), the symptom began at the stage to which it was provisionally assigned, if it began at an earlier or a later stage (and which one), or if it was absent altogether (i.e., the respondent did not recognize that symptom as ever having been experienced over the course of the patient's illness to date). We assumed that respondents for patients who were earlier in the course of their illness would not recognize most symptoms assigned provisionally to later PPA stages; moreover, we wished to avoid causing distress by confronting respondents with symptoms they might not have anticipated. Respondents were therefore able to discontinue this first section of the survey at any point. The point at which the respondent discontinued this section of the survey was taken to indicate that patient's current PPA stage. Participants were able to review and edit their responses at any point via a “Back” button.

In the next section of the survey, respondents were presented with a representative list of symptoms present (1) in the other PPA variant (sampling each of the domains A, B1, and B2), and (2) (principally as an internal “control,” to asses response bias) in a staging system for a clinically distinct, “visual” dementia (posterior cortical atrophy [PCA])^36^: for each of these symptoms, caregivers were again asked to indicate whether the symptom was present, and if so, to which stage it should be assigned. They were additionally given the opportunity to make additional comments about symptoms not covered elsewhere in the survey, and their impressions of the staging system in its current form, for the purpose of qualitative analysis.

### 2.3 Validation of diagnosis

To allow us to estimate the overall validity of syndromic diagnoses as listed by caregivers in the consolidation survey, survey respondents recruited from the UK PPA Support Group were given the option of including their name when they completed the survey. When this was volunteered, we were able to cross‐check whether that caregiver–patient dyad had previously participated in the PPA research program at Queen Square and if they had, to check that the diagnosis listed in the survey for that person living with PPA was corroborated by detailed neuropsychological and neuroimaging data in the research database.

### 2.4 Analysis of clinical and demographic data

Clinical and demographic data were analyzed using JASP version 0.16.2 and Stata version 14.2. Groups (i.e., variants) were compared using analyses of variance for continuous variables and Fisher exact tests for categorical variables.

### 2.5 Quantitative analysis of survey responses

For each symptom in the consolidation survey, we calculated the percentage of respondents who had declared that symptom to be “present,” regardless of PPA subtype or stage; a symptom was retained only if a majority (at least 50%) of caregivers who provided a response to a given symptom reported it was present at some stage. In addition, we calculated the percentage of respondents who considered each symptom had been assigned to the correct stage. If a majority of respondents considered a symptom should be reassigned to an earlier or later stage, it was reassigned accordingly. When a symptom was assigned to more than one stage (e.g., the majority was tied across two stages), it was retained only at the earlier stage for which it first achieved criterion (because in general, the earliest appearance of a symptom is most informative for signaling disease progression and/or planning care needs). We assessed the “confidence” of stage assignment for each symptom as the proportion of respondents for that symptom in agreement with the final stage to which the symptom was assigned.

### 2.6 Qualitative analysis of survey responses

Caregiver comments on the exploratory and consolidation surveys were analyzed qualitatively using framework analysis.^38^, ^39^ A tentative framework was proposed by one of the authors (C.J.D.H.) after familiarization with the whole dataset. This initial coding framework was then applied to a subsample of 20% of the dataset, which was then reviewed by another author (E.H.); discrepancies or differing interpretations were reviewed and discussed. Based on this consensus, a thematic framework was developed using tables of data in Microsoft Excel (v2016) and applied to the full survey dataset.

### 2.7 Generation of a prototype functional impairment scale for PPA

**FIGURE 2:**
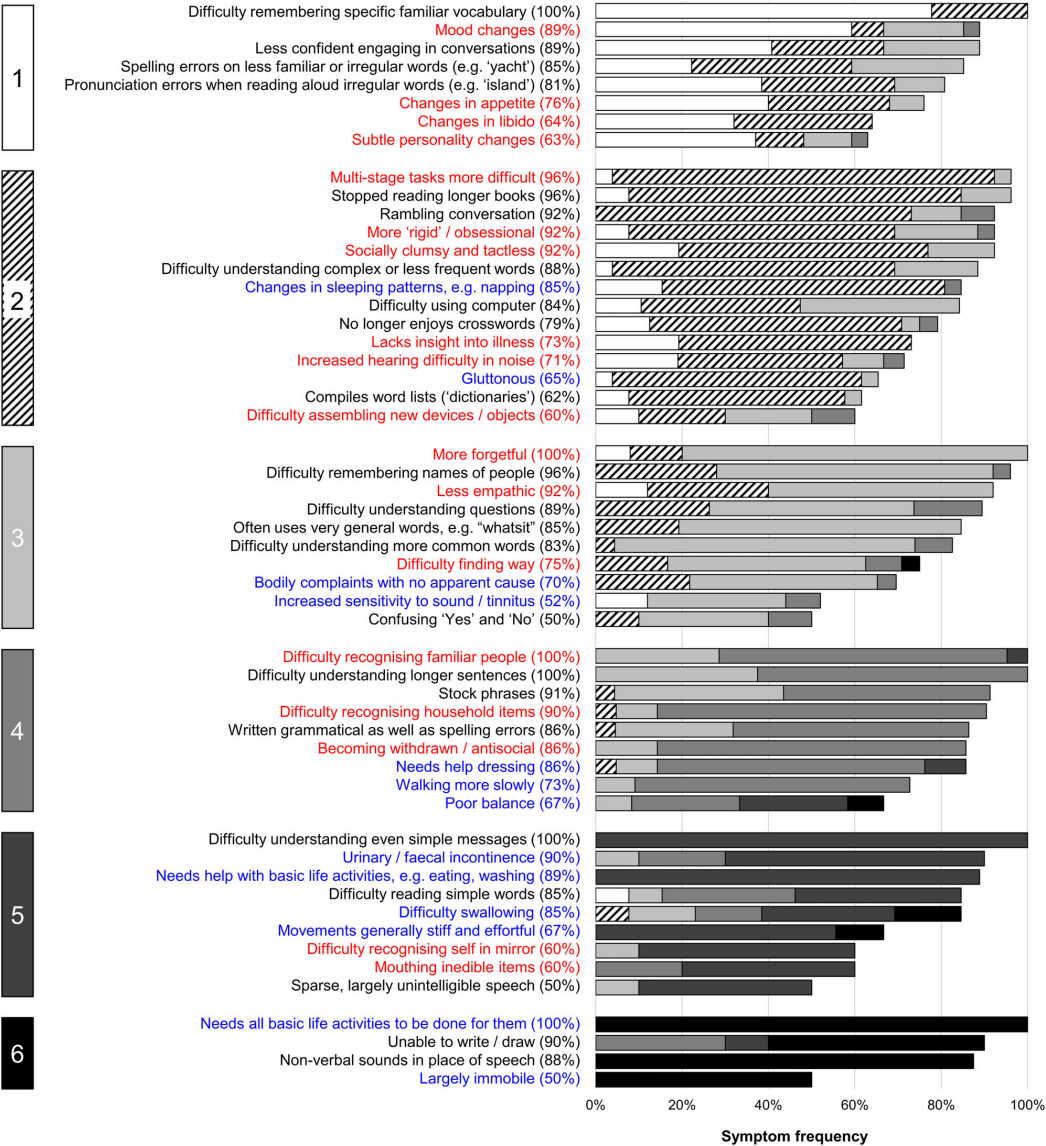
Symptom frequencies and confidence in symptom placement by stage for semantic variant primary progressive aphasia. The figure shows all symptoms included in the caregiver survey for semantic variant primary progressive aphasia. A symptom was retained only if a majority (at least 50%) of caregivers who provided a response to a given symptom reported it was present at some stage of the illness (see Table S2 in supporting information for complete symptom list). Boxes on the left‐hand side denote stages, numbered 1 (least severe) to 6 (most severe). Written symptom labels are color‐coded based on domains of verbal communication (A = black) and non‐verbal functioning (B1 = non‐verbal thinking and personality, red; B2 = personal care and well‐being, blue). Horizontal bars indicate the “confidence” of symptom staging, calculated as the percentage of people responding to a given symptom who endorsed placement of that symptom in its final stage (i.e., the highest agreement achieved for placement of that symptom). Symptoms have been ordered within stages in descending order of overall frequency. Key “milestone” symptoms from this figure have been used to generate the prototype functional impairment scale for svPPA (the PPA‐Squared scale, see Table 3): clinical stages 1 to 3 here generated symptoms corresponding to scale levels 1 to 3, while clinical stages 4 to 6 all contributed symptoms to the “severe” scale level 4. This scaling of clinical stages allows more fine‐grained grading of earlier stage disease (when opportunities for intervention are greatest) while acknowledging that the incremental impact on daily life function between successive clinical stages becomes less defined once illness is already advanced. PPA‐Squared, Primary Progressive Aphasia Progression Planning Aid; svPPA, semantic variant primary progressive aphasia

**FIGURE 3:**
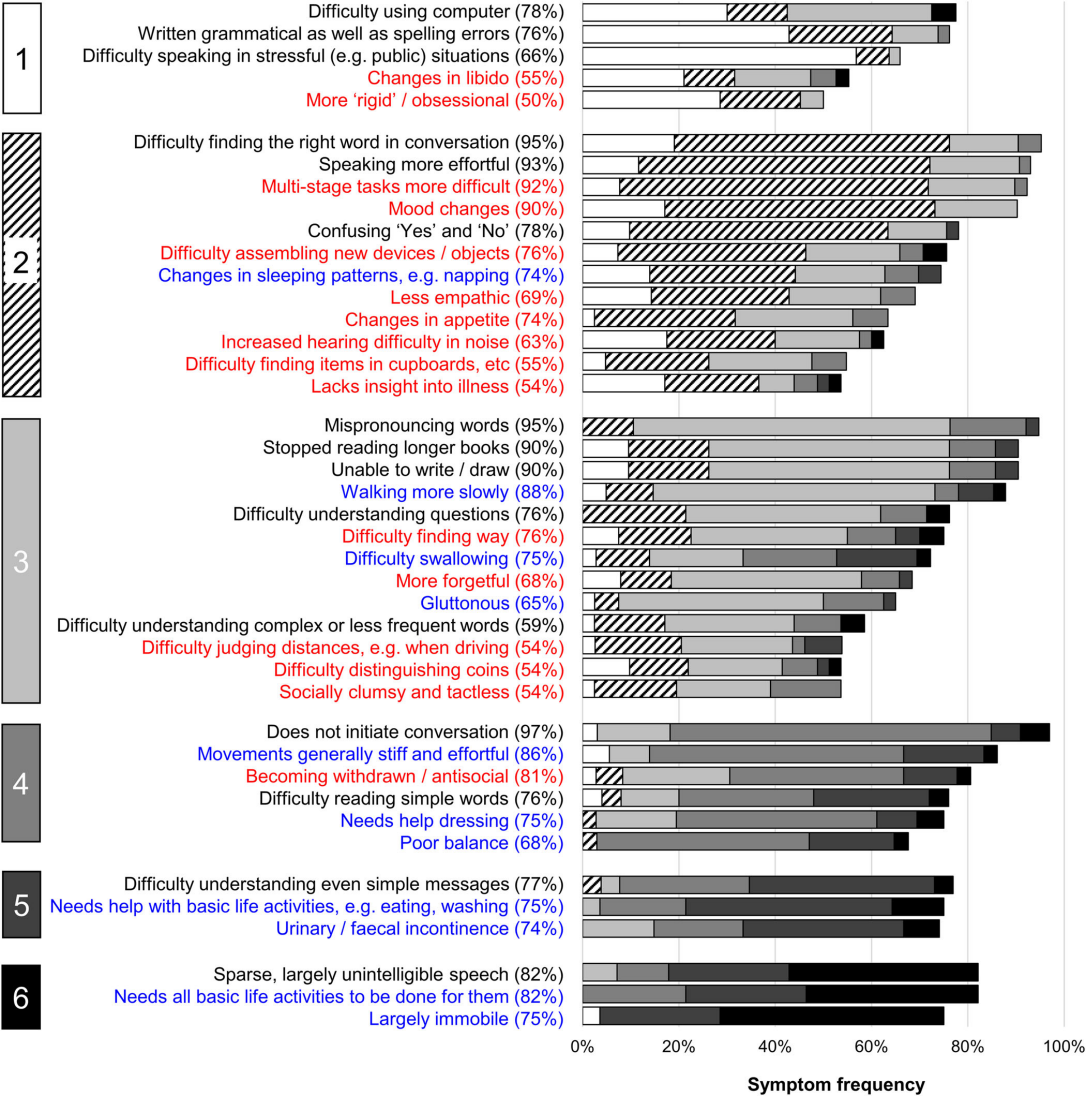
Symptom frequencies and confidence in symptom placement by stage for non‐fluent/agrammatic variant primary progressive aphasia. The figure shows all symptoms included in the caregiver survey for non‐fluent/agrammatic variant primary progressive aphasia. A symptom was retained only if a majority (at least 50%) of caregivers who provided a response to a given symptom reported it was present at some stage of the illness (see Table S3 in supporting information for complete symptom list). Boxes on the left‐hand side denote stages, numbered 1 (least severe) to 6 (most severe). Written symptom labels are color‐coded based on domains of verbal communication (A = black) and non‐verbal functioning (B1 = non‐verbal thinking and personality, red; B2 = personal care and well‐being, blue). Horizontal bars indicate the “confidence” of symptom staging, calculated as the percentage of people responding to a given symptom who endorsed placement of that symptom in its final stage (i.e., the highest agreement achieved for placement of that symptom). Symptoms have been ordered within stages in descending order of overall frequency. Key “milestone” symptoms from this figure have been used to generate the prototype functional impairment scale for nfvPPA (the PPA‐Squared scale, see Table 3): clinical stages 1 to 3 here generated symptoms corresponding to scale levels 1 to 3, while clinical stages 4 to 6 all contributed symptoms to the “severe” scale level 4. This scaling of clinical stages allows more fine‐grained grading of earlier stage disease (when opportunities for intervention are greatest) while acknowledging that the incremental impact on daily life function between successive clinical stages becomes less defined once illness is already advanced. nfvPPA, non‐fluent/agrammatic variant primary progressive aphasia; PPA‐Squared, Primary Progressive Aphasia Progression Planning Aid

We used the clinical stages defined in the caregiver surveys to derive a prototype functional impairment scale (the PPA Progression Planning Aid, “PPA‐Squared”) for svPPA and nfvPPA, incorporating key symptoms relevant to daily life functioning over the course of the illness. Symptoms comprising this scale were categorized under the same three broad functional domains presented in Figures 2 and 3 (communication; non‐verbal thinking and personality; personal care and well‐being). Symptoms were selected on the basis that they signaled significant illness transitions or “milestones” relevant to occupational and social activities, personal needs, and other important aspects of daily life functioning. Ordering of symptoms was guided by the six‐stage survey staging scheme; however, in addition to symptom progression the scale was designed to capture changes in symptom severity that might develop at varying points in the illness for different functional domains and in different individuals. Thus, we organized the PPA‐Squared scale under different “levels” of symptom severity, allowing each functional domain to be scored separately. The correspondence of these levels to the overall illness stages from the survey is further elaborated below. In addition, mindful of future clinical applications, in compiling the scale we distilled the original symptom descriptors (as presented to caregivers; see Table S1) to succinct labels.

### 2.8 Ethical approval

Data for this study from UK PPA Support Group members were collected under the Rare Dementia Support (RDS) Impact Study protocol, which has been published separately.^40^ Ethical approval was granted by the University College London Research Ethics Committee (8545/004: Rare Dementia Support [RDS] Impact Study). Additional local site approval for support group members in Sydney was granted by the South Eastern Sydney Local Health District HREC (2020/ETH02530). All survey respondents gave informed consent, in accordance with Declaration of Helsinki guidelines. Survey data were downloaded from the online platform and stored within the University College London Data Safe Haven to protect against unauthorized access.

### 2.9 Data availability

The data that support the findings of this study are available on request from the corresponding author. The data are not publicly available as they include information that could compromise the privacy of the research participants.

## 3 RESULTS

The final stages are presented in Figure 2 for svPPA and Figure 3 for nfvPPA. The raw data supporting the stage assignments are presented in Tables S2 (for svPPA) and Table S3 (for nfvPPA). Demographic and clinical characteristics of patients whose data were provided for the caregiver consolidation survey are summarized in Table 1. Themes, subthemes, and illustrative caregiver comments from the qualitative framework analysis are presented in Table 2. Prototype functional impairment rating scales for svPPA and nfvPPA are given in Table 3.

**TABLE 1:** Breakdown of general respondent characteristics in the caregiver consolidation survey.

	svPPA	nfvPPA
UK cohort (*n*)	24	33
Australian cohort (*n*)	3	13
All survey respondents (*n*)	27	46
Bereaved respondents (*n*)	1	10
Relationship status (partner/other, *n*)	19/8	34/12
Age at which first symptom noticed	62.59 (6.29)^b^	65.08 (8.18)^b^
Age at first GP appointment	64.46 (6.31)^c^	66.20 (8.36)^b^
Delay seeking medical advice (years)	1.90 (1.84)^c^	1.13 (1.57)^b^
Age at diagnosis	65.93 (6.36)^b^	67.64 (8.40)^c^
Time to diagnosis (years)	3.34 (2.51)^b^	2.30 (2.22)^d^
Age at survey	70.61 (3.45)^e^	71.57 (8.17)^f^
Symptoms presented in caregiver survey (*n*)	75	62
Symptoms included in final staging list (*n*)	54	44
Stage at time of survey (*n*):		
Stage 1	0	0
Stage 2	0	2
Stage 3	3	6
Stage 4	13	11
Stage 5	2	6
Stage 6	9	21

*Note*: Mean (standard deviation) data are presented unless otherwise specified. Not all questions were answered by all respondents, and missing data are coded as follows: ^a^n‐1; ^b^n‐2; ^c^n‐3; ^d^n‐4; ^e^n‐5; ^f^n‐18. The larger number of missing responses to the “Age at survey” question was largely accounted for by bereaved caregivers, that is, the person they were answering the survey about was already deceased.

Abbreviations: GP, general practitioner; nfvPPA, non‐fluent/agrammatic variant primary progressive aphasia; svPPA, semantic variant primary progressive aphasia.

**TABLE 2:** Qualitative framework analysis: Themes, subthemes, and illustrative caregiver comments.

Theme/Subtheme	Illustrative caregiver comments	Diagnosis
**Theme 1: Impact and experience of symptoms**		
Emotional impact of the condition	“The emotional and social withdrawal were the hardest aspects to deal with as spouse.”	nfvPPA
Earliest symptoms noticed	“Compulsive/impulsive behaviors seemed an early stage symptom, as well as socially inappropriate actions, with my wife often believing them to be humorous.”	nfvPPA
	“The first thing we really noticed was being unable to read a book, used to read 2–3 per week and inability to follow television programs, as well as using the same word repeatedly in conversation.”	nfvPPA
Adding additional information about symptoms already listed in the stages	"I find it helpful to think about the sort of sentences mum wouldn't understand (so to give examples what the language difficulties are like since language is the primary issue) when explaining to people. So, for example, to think, if I said ‘mum, can you bring me a plate?’ she wouldn't understand that as a sentence initially, but if she grasped the word plate (either because I point/on this occasion the word is familiar) she would know what I meant (i.e., the concept of the sentence makes sense to her but she hasn't computed the meaning of words).”	svPPA
	“I'm not sure if my husband's personality changes are typical or unusual. He has had problems with facial recognition for 2 years … this leads him to think he knows people when he doesn't and therefore to strike up a conversation with strangers and appear very amiable and chatty—even if I know the content of the conversation is inaccurate or repetitive (because the content of his chatter is usually much the same).”	svPPA
Adding descriptions of symptoms not included	“I have noticed that there is a tendency to do half a job. For example, when drying after a shower there is a failure to dry the whole body and the back remains wet. When shampooing hair, the shampoo will go on but will not be washed off. … I believe that we are at the moderate stage at present.”	nfvPPA
**Theme 2: Illness progression/trajectory**		
Fluctuations in decline	“One thing that has been very noticeable with my wife's condition is that changes happen very quickly sometimes from one day to the next. It is not a gradual decline. However, once the drop off has happened I have on occasions been able to get my wife to recover some of the loss. It appears to me as though the brain is trying to find other ways of trying to get around the problem that it has been faced with.”	nfvPPA
	“There seems to be no recognition of the ‘rollercoaster’ nature of FTD/PPA, which is rarely gradual in our (collective) experience, with stops and starts, regressions, plateaux and also improvements.”	nfvPPA
Speed of progression	“The progression seemed faster than for other people we came across with this illness. For us it was 3 years from diagnosis to death.”	nfvPPA
	“This is a very difficult journey. Things seem to be accelerating now but have no idea of what is to come.”	svPPA
**Theme 3: Experience of doing the research**		
Difficulties answering questions on behalf of the plwPPA	“These points are from pure observation since my wife has not been able to speak since the moderate stage of the condition and when she could speak she would never accept there was anything wrong with her.”	nfvPPA
	“Just to say that I did find it quite difficult to recall the detail as my wife's PPA has progressed and as a consequence I needed to make a sometimes not too educated guess about the period of time that my wife has spent on each stage.”	svPPA
Difficulties with the way the survey was designed	“I found it difficult to complete this questionnaire, not necessarily for emotional reasons, more because of the requirement to allocate the ‘correct’ positioning of the various symptoms to a particular stage.”	nfvPPA
	“The ideal way to fill in this form would have been to refer to a diary. Unfortunately, I never kept one, which looking back is a regret.”	svPPA
**Theme 4: Utility of the stages**		
Perceived strengths of the stages	“This is SO USEFUL and helpful for others as they embark on this challenging journey. Even if everyone experiences different symptoms at different rates, it's so helpful as a carer to read and realize/understand that these are ‘normal/expected’ with these diagnoses … you are not alone, weird, or must feel ashamed. Thank you—hugely appreciated from a carer's point of view—so much if not all of it lands squarely on our shoulders!”	svPPA
	“The stages so far are what I have experienced, no one ever told me the likely stages, it is left to carers to search for stages. This makes it very difficult to cope with and to prepare for. Describing the stages is a good idea and will be helpful for many carers.”	nfvPPA
Perceived limitations of the stages	“Once the ability to communicate is lost it is difficult to know what stage the person is in, apart from visible or physical signs.”	nfvPPA
	“Stages make it sound very organized and predictable but, unlike Alzheimer's perhaps, the progression of PPA isn't like that. You can't predict what will happen next or when it will happen, only that it is likely to happen some time. It sounds very harsh but this is a cruel disease.”	nfvPPA
**Theme 5: Suggestions for further development/dissemination**		
Incorporating care milestones into the stages	“To then match the stages to actions which may be required by the patient and their carers, for example, to sort out legal matters, wills, powers of attorney, guardianship, advanced care directives and so on, at the earliest possible stages. When to stop driving, when assistance with self‐care, assistance with activities of daily living, 24 hour care etc. may be required (rough guides accounting for individual variations, valuable to carers nonetheless…).”	nfvPPA
Aligning stages with intact abilities	“I wonder if it might be useful to find out what people can do at different stages for the different dementias rather than what is declining … not so interesting for you medically speaking but very useful sharing information for carers. Making things meaningful and purposeful for both carer and caree is so important and may make life more meaningful/rewarding/satisfying.”	svPPA
Importance of how and when information is accessed	“I think the points made in the introduction are very valid—a road map of symptoms presented at an early stage could well be overwhelming and distressing to contemplate. A partner may feel unequal to the task of managing these symptoms when they are described in behavioral terms. The person with the diagnosis may feel life would not be worth living with these symptoms.”	svPPA
	“I have thought hard about whether it would be useful to inform people that they do not have to read all the stages but limit themselves to the stage they think the person is at. I think both of us would have been quite devastated and found it hard not to become depressed if we had had all the stages mapped out as will now be available. We have found it easier to deal with things as they came up. It certainly would have been very helpful for me, especially as the carer, to know what the next stage would entail but I would not have wanted to go further. I understand that this is a personal view and not everyone will think the same. Some may want to know the whole of what is to come, but that should be a conscious decision not one done accidentally.”	svPPA

*Note*: The table presents themes and subthemes identified in the qualitative framework analysis, with illustrative caregiver quotations representing each subtheme.

Abbreviations: FTD, frontotemporal dementia; nfvPPA, non‐fluent/agrammatic variant primary progressive aphasia; plwPPA, person living with primary progressive aphasia; PPA, primary progressive aphasia; svPPA, semantic variant primary progressive aphasia.

**TABLE 3:** A prototype functional impairment scale for primary progressive aphasia, the Primary Progressive Aphasia Progression Planning Aid (PPA‐Squared).

svPPA					
Domain	Level 0: Presymptomatic	Level 1: Very mild	Level 2: Mild	Level 3: Moderate	Level 4: Severe
**Communication**	No changes	Specific (e.g., occupational) vocab may not be understood Spelling errors (irregular words)	Less common words not understood	Common words not understood	Speech stereotyped, becoming unintelligible
**Nonverbal thinking and personality**	No changes	Mood, appetite, libido changes	Multi‐step tasks more difficult More socially clumsy Lacks insight into illness Increased hearing difficulty in noise	More forgetful Navigation affected	Recognition of familiar people, household items affected Social withdrawal
**Personal care and well‐being**	No changes	No changes	Sleep changes Gluttonous	Bodily complaints without explanation Sound sensitivity/tinnitus	Dressing and other basic life activities require help Incontinence Swallowing difficulty Walking, balance affected

nfvPPA					
Domain	Level 0: Presymptomatic	Level 1: Very mild	Level 2: Mild	Level 3: Moderate	Level 4: Severe
**Communication**	No changes	Grammatical and spelling errors Speaking difficulties in stressful (e.g., public) situations	Conversational word‐finding difficulty Speaking more effortful Confusing “yes” and “no”	Mispronouncing words Writing/drawing no longer possible Questions may not be understood Less common words not understood	Speech sparse, becoming unintelligible Simple messages not understood
**Non‐verbal thinking and personality**	No changes	Libido changes More “rigid”, obsessional	Multi‐step tasks more difficult Mood, appetite changes Less empathic Increased hearing difficulty in noise Lacks insight into illness	Navigation affected More forgetful Visuospatial judgment affected Socially clumsy	Social withdrawal
**Personal care and well‐being**	No changes	No changes	Sleep changes	Walking slower Swallowing difficulty Gluttonous	Movements stiff and effortful Dressing and other basic life activities require help Incontinence Poor balance

*Notes*: The table summarizes key symptoms relevant to daily life functioning that emerge over the clinical course of semantic variant primary progressive aphasia (top) and non‐fluent/agrammatic variant primary progressive aphasia (bottom), as identified from caregiver surveys. Symptoms are categorized under the three broad functional domains used when collecting data from caregivers (see text, Figures 2 and 3). Symptoms within each cell are listed in order of frequency (most frequent at the top), as endorsed in caregiver surveys (see Figures 2 and 3). The scale is designed to be used as a metric for illness progression and/or severity: a clinician would administer the scale to patient and caregiver as part of a clinical interview and for each domain, the level of impairment would be scored for each domain separately, and combined to generate a total score. Thus, while there is an overall correspondence between increasing “levels” here and the “stages” presented in Figures 2 and 3, an individual patient might score (for example) “level 2” communication symptoms but “level 1” non‐verbal thinking and personality symptoms, and so on (see text). Note that the scale is provisional pending further validation (see text).

Abbreviations: nfvPPA, non‐fluent/agrammatic variant primary progressive aphasia; svPPA, semantic variant primary progressive aphasia.

### 3.1 Demographic and clinical characteristics

The survey was accessed a total of 206 times. Respondents included 37 caregivers for people with lvPPA, not further considered here. In addition, data were excluded for the following (not mutually exclusive) reasons: no response provided beyond agreeing to take part, *n* = 84; respondent indicated that the person for whom they were answering the survey did not have a canonical PPA variant, *n* = 3; data directly duplicated a previous response, *n* = 3; the respondent was a professional caregiver, *n* = 1; and/or the person completing the survey had a diagnosis of PPA themselves, *n* = 7. After exclusions, responses were received from 27 primary caregivers acting for people with svPPA, and 46 for people with nfvPPA.

Diagnostic groups for surveyed respondents did not differ significantly in age at symptom onset (F[1,69] = 1.34, P = 0.252), age at first visit to general practitioner (GP; F[1,68] = 0.63, *P* = 0.431), delay seeking medical advice (calculated as age at first visit to GP minus age at symptom onset; F[1,67] = 1.74, *P* = 0.192), age at diagnosis (F[1,67] = 0.46, *P* = 0.502), time to diagnosis (calculated as age at diagnosis minus age at symptom onset; F[2,100] = 1.67, *P* = 0.196), age when survey completed (F[1,46] = 0.24, *P* = 0.624), relationship status (Fisher exact *P* = 0.790), or stage at the time of the survey (Fisher exact *P* = 0.294). The proportion of bereaved caregivers across diagnostic groups did differ significantly (Fisher exact *P* = 0.046), which was driven by a larger number of bereaved respondents in the nfvPPA group (*n* = 10) compared to the svPPA (*n* = 1) group. In both syndromes, the illness tended to declare itself from the early to mid‐seventh decade, and with substantial individual variation (Table 1). For 24 self‐identified respondent caregivers of people with PPA also participating in the Queen Square research program, review of available neuropsychological and neuroimaging data in the research database confirmed the syndromic diagnosis listed in the survey in all cases. The average delay from symptom onset to diagnosis was well over 2 years in both PPA syndromes, albeit with a wide range in each syndrome.

### 3.2 Quantitative analysis of survey responses

More symptoms were endorsed overall at the frequency criterion (≥ 50%) by caregiver respondents for svPPA (54 symptoms) than for nfvPPA (44 symptoms). However, the six‐stage framework (ranging from stage 1, “Very mild” to stage 6, “Profound”; Table S1) was endorsed in each PPA syndrome, and the overall profile of symptom development across domains was broadly similar for both syndromes. In both syndromes, symptoms relating to communication and non‐verbal conduct and well‐being were present at stage 1 and symptoms relating to non‐verbal thinking by stage 2. Earliest symptoms included erosion of specific vocabulary in svPPA, and difficulty conversing in stressful situations, and binary (e.g., “yes”/“no”) reversals in nfvPPA; spelling errors were an early feature in both syndromes. Increased hearing difficulty in noise also developed early in both syndromes. Changes in inter‐personal behavior (e.g., altered libido) were also among the earliest non‐verbal features in both syndromes, and loss of insight was also endorsed for both syndromes. Problems with episodic memory, route finding, praxis, and task sequencing developed in both syndromes by stage 3. Of 15 additional “control” symptoms relevant to PCA presented in the survey, four (reflecting non‐verbal parietal lobe functions, i.e., relating to praxis and visuoperceptual awareness) were endorsed by caregivers for inclusion in the stages for a PPA syndrome (most frequently in nfvPPA; details in Tables S2 and S3). Physical symptoms began in stage 3 for both nfvPPA and svPPA and stage 4; the nature of these first physical symptoms varied between syndromes, with patients with nfvPPA developing difficulties moving and swallowing, and patients with svPPA unexplained somatic complaints, hyperacusis, and tinnitus. The syndromic specificity of symptoms diminished over the course of the illness (examining all symptoms listed in stages 1 and 2 in both syndromes, 49% were unique to one variant, whereas this was the case for just 37% of symptoms listed in stages 5 and 6). End‐stage PPA in both syndromes was characterized by vocal production limited to sparse, non‐verbal sounds, inability to understand others, immobility, and complete dependency for basic activities of daily life.

There was a wide range of “confidence” in symptom placement, across stages and syndromes (Figures 2 and 3; Tables S2 and S3). For svPPA, overall respondent agreement with symptom placement increased over stages (mean 55% in stage 1, 89% in stage 6). In contrast, for nfvPPA, overall respondent agreement with staging remained moderate across stages (mean 53% in stage 1, 51% in stage 6).

### 3.3 Qualitative analysis of survey responses

The qualitative framework comprised five major themes (Table 2): (1) impact and experience of symptoms, (2) illness progression/trajectory, (3) experience of doing the research, (4) utility of the stages, (5) suggestions for future development/dissemination. Thirteen subthemes were identified within these major themes, and together themes and subthemes encompassed respondents’ experiences of living with PPA and of the staging survey, and their suggestions for implementation.

### 3.4 Features of the prototype functional impairment scale for PPA

Analysis of survey data revealed that the illness journey through stages 1 to 6 was clearly not linear; indeed, by the time a person reached stage 4, their impairment was likely already to be debilitating, meaning that the functional “step down” between stages 4 to 5, for example, was less substantial than that between stages 1 to 2. In generating the prototype functional impairment scale for PPA, PPA‐Squared (Table 3), we therefore collapsed across stages 4 to 6 for the level of most marked functional impairment, to arrive at a five‐level scale of illness progression and severity: (0, presymptomatic; 1, very mild; 2, mild; 3, moderate; 4, severe).

Separable (but partially convergent) profiles of “milestone” symptoms were identified for each of the PPA syndromes surveyed here. Early illness stage/mild milestone symptoms included syndrome‐specific communication dysfunction (loss of vocabulary in svPPA, loss of conversational fluency and “yes”/“no” confusions in nfvPPA) but also non‐verbal behavioral changes (appetite changes in svPPA, mental “rigidity” and obsessionality in nfvPPA), while reduced insight was a common early symptom in both syndromes. Milestone symptoms associated with somewhat later stage/more severe disease included difficulties understanding everyday words in svPPA, and emergence of walking and swallowing difficulties in nfvPPA. End‐stage/most severe disease in both syndromes manifested with (besides loss of communication function) fundamental problems with personal care and well‐being, in the context of impaired motor functions and incontinence.

## 4 DISCUSSION

Here we have presented a staging scheme for symptom development in svPPA and nfvPPA based on the lived experience of patients’ caregivers. From this, we derived milestone symptoms signaling significant illness transitions that constitute a progression and severity scale of daily‐life functional impairment in these diseases, the PPA‐Squared scale. We hope these will serve as complementary tools for people living with PPA, their caregivers, and clinicians. The clinical staging scheme (Figures 2 and 3) offers a databank with granular detail about symptom diversity and frequency in svPPA and nfvPPA, and how symptoms tend to cluster at a given illness stage. The PPA‐Squared distills this granularity: it is oriented toward metrics that might allow the clinician to quantify where a patient is in their illness journey, and thereby guide management and care decisions. This might be applied to PPA somewhat analogously to the Clinical Dementia Rating scale in AD and other dementias,^41^ with a view in particular to the generation of outcome measures for clinical trials. In both PPA subtypes (corroborating previous findings^4^, ^5^), respondents reported an average delay over 2 years from symptom onset to diagnosis, arguing that we need timelier characterization of earliest stage disease to facilitate trial entry. PPA‐Squared reflects this clinical imperative: it scales clinical stages to allow more fine‐grained grading of earlier stage, milder illness (clinical stages 1–3 are captured in PPA‐Squared levels 1–3), while acknowledging that the incremental functional impact of successive clinical stages is less defined once illness is already advanced (clinical stages 4–6 are subsumed as “severe” under PPA‐Squared level 4). Individual patients will move through the clinical stages of PPA differently (see Figures 2 and 3): PPA‐Squared is accordingly designed to score levels of impairment under each of the three surveyed major functional domains (communication; non‐verbal thinking and personality; personal care and well‐being) separately, combining these to generate a total scale score. We emphasize, however, that this scale is a proof‐of‐principle prototype that awaits further refinement and validation; its clinical utility must therefore be considered provisional.

Our findings paint a complex picture of PPA evolution, endorsing previous work.^6^, ^7^, ^10^, ^42^, ^43^, ^44^, ^45^, ^46^, ^47^, ^48^, ^49^ Both subtypes here were led by partially syndrome‐specific communication impairments: svPPA, by symptoms consequent to loss of vocabulary, and nfvPPA, by dysfluency manifesting in situations in which there is a “performance” requirement (e.g., speaking in public). Certain syndromic hallmarks (e.g., compiling personal “dictionaries” in svPPA, binary reversals in nfvPPA) were identified. Contrastingly, spelling errors developed early in both syndromes. Partial syndromic specificity was also evident for non‐verbal functional abnormalities; while earliest socio‐emotional behavioral alterations took different forms between syndromes (in svPPA, appetite changes; in nfvPPA, mental rigidity), hearing changes occurred early in both syndromes. Syndromes tended to converge clinically, physical (especially motor and autonomic) neurological impairments becoming more salient longitudinally. However, even at later stages, the imprint of variant‐specificity was evident: swallowing and mobility problems developed earlier in nfvPPA, while difficulty recognizing familiar people and household items characterized svPPA.

This work highlights several clinical issues. Reliable detection of earliest symptoms would facilitate timelier and more accurate diagnosis: particularly apposite given the delay between onset and diagnosis. The factors that influence this delay need elucidation. Early features of PPA syndromes here included both verbal phenomena (e.g., binary reversals^50^) and non‐verbal phenomena (e.g., impaired hearing, and changes in libido, sleep, empathy, and insight) that are not recognized in consensus criteria.^1^ The prevalence of non‐verbal features in stage 1 challenges the presumption that language deficits lead the clinical onset of PPA.^1^ Equally, we lack tools for the comprehensive assessment of needs and evidence‐based interventions in advanced PPA. The symptom sequences shown in Figures 2 and 3 (Tables S2 and S3) should enable better clinical prognostication in individual patients, and inform development of care pathways and customized interventions targeted earlier at specific PPA subtypes: a window of opportunity often missed.^51^, ^52^, ^53^ However, given the variable rates at which patients with PPA progress (Table 2) and the variation of symptom clusters within stages (Figures 2 and 3), mapping personalized illness trajectories will be crucial in defining care priorities and guiding, for example, statutory assessments.

Neurobiologically, our findings support recent work emphasizing multidimensional phenotypic overlap in PPA, arising from distributed brain network alterations.^28^, ^29^, ^54^, ^55^ Our findings also underline the importance of the temporal dimension in interpreting the phenotypic spectrum of PPA. In svPPA, the sequence of symptoms reflects involvement of left followed by right temporal lobes and their connections: the “semantic appraisal” network targeted by svPPA supports a range of cognitive and behavioral functions,^56^, ^57^, ^58^, ^59^ while “extra‐temporal” (e.g., motor) symptoms occur late. By contrast, the sequence of multidimensional symptoms in nfvPPA suggests early involvement of motor control networks including basal ganglia, supporting neuroanatomical studies.^16^, ^45^ The varying “confidence” in symptom staging is also informative (Figures 2 and 3; Tables S2 and S3): consensus was highest for svPPA, endorsing the view that this is a highly coherent syndrome, whereas nfvPPA is more heterogeneous.^3^, ^8^, ^29^, ^54^, ^60^, ^61^, ^62^

While our methodology builds on recent online data collection initiatives,^63^, ^64^ it has limitations. Caregiver reports were retrospective—this is not necessarily disadvantageous (subtle symptoms may not initially be recognized as such) but is open to recall bias, and may have been compounded by an over‐representation of bereaved caregivers for people with nfvPPA. The present stage divisions and criteria for symptom inclusion are pragmatic but arbitrary. Certain symptoms might be specific for particular PPA syndromes yet develop infrequently. Symptoms here were not quantified in frequency/intensity, and symptom descriptors were often broad (e.g., “difficulty using a computer,” is likely underpinned by different deficits between syndromes). This is particularly relevant to complex socio‐emotional behavioral changes such as “mental rigidity” or “lack of empathy.” It is unclear how the proposed stages map onto objective disease progression measures, or indeed, whether six stages are optimal; fewer stages would provide greater uniformity within syndromes but lose granularity. The present framework lacks information on the duration of particular stages or overall disease tempo: clinical experience suggests that individual temporal variability is wide, a point corroborated by qualitative analyses (Table 2).

Moreover, the present cohort does not represent the full PPA spectrum. Respondents were all English‐speaking members of support groups with internet access. It is unclear how PPA‐Squared would be recapitulated in linguistically and socio‐culturally diverse populations. Even within the surveyed cohort, qualitative responses highlighted varying opinions on the utility, relevance, and impact of PPA‐Squared (Table 2). Finally, we focused on two canonical PPA syndromes but many PPA patients do not meet consensus criteria for these.^3^, ^6^, ^19^ Furthermore, there remains a need to assess whether symptom progression in lvPPA is adequately characterized by staging scales developed for AD.

These caveats suggest directions for future work. Findings should be extended and validated in larger, more diverse cohorts. Longitudinal studies are required to ensure complete stage coverage without recall bias, to define temporal dynamics of stage transitions, and to capture the full range of discrete functional milestones (e.g., use of mobility aids, introduction of assisted feeding) that guide care decisions.^65^ Broad symptom categories could be deconstructed and more fine‐grained information collected about daily‐life impact. Ideally, first‐person patient perspectives would be gathered alongside caregivers’, to capture experiential phenomena (e.g., hallucinations) that may elude third‐person recording, and to better characterize loss of insight. The clinical staging scheme (Figures 2 and 3) and the PPA‐Squared (Table 3) focus on deficit and disability; losses should be contextualized by documenting retained capacities (Table 2). Ongoing engagement with lay stakeholders and clinicians should further critique the acceptability and utility of this work.^66^ PPA‐Squared should be correlated with neuropsychological scores, structural and functional neuroanatomy, and laboratory indices,^67^ as well as clinical rating scales;^12^, ^25^, ^57^, ^68^ it could also facilitate computational disease progression modelling approaches.^69^, ^70^ *Post mortem* data would link clinical symptoms to neuropathology, while genetic cohorts represent an opportunity to unpack earliest disease stages (i.e., “PPA‐Squared 0”).^71^

Functional illness staging and grading of daily‐life symptoms affecting communication and other domains^72^ should support care decisions and interactions between clinical teams, as well as providing outcome measures for trials in PPA and other dementias with prominent language features.^73^ Further development of the stages and scale will therefore entail collaborative validation and distillation of large datasets. We hope this work will motivate international collaborations to take these next steps, importantly including non‐English languages (see, e.g., a Spanish translation of PPA‐Squared in Table S4 in supporting information). Our findings suggest that a fresh consensus on PPA diagnostic criteria and management guidelines may be timely, to incorporate syndromic hallmarks such as binary reversals in nfvPPA and auditory and non‐verbal behavioral features across subtypes. We envisage that particular symptoms might inform development of diagnostic “stress tests” for PPA, based on conversation analysis^74^ or communication in multi‐talker environments,^47^ facilitating early detection. The framework adopted in the present study for engaging the expertise of people with lived experience of PPA potentially has translational relevance to the much wider dementia population, and could help shape more inclusive research initiatives and public health policy. However, the overriding value of the PPA clinical staging scheme and PPA‐Squared scale will lie in the personalized care of individuals—to signpost their journey, harness support and treatments, and help them plan for the future.

## CONFLICT OF INTEREST STATEMENT

C.H. reports funding support for the present manuscript from the Royal National Institute for Deaf People, Dunhill Medical Trust, Wellcome Trust, and National Institute for Health and Care Research. B.T. reports funding support for the present manuscript from the Economic and Social Research Council. E.H. reports funding support for the present manuscript from the Economic and Social Research Council, and the National Institute for Health Research Dementia Research Initiative. J.J. reports funding support for the present manuscript from the National Brain Appeal. N.K. reports funding support for the present manuscript from the Alzheimer's Society. K.Y. reports funding support for the present manuscript from the Alzheimer's Society and National Institutes of Health; additional funding not related to the present manuscript from the National Brain Appeal; and personal fees from Roche outside the submitted work. C.M. reports funding support for the present manuscript from the National Institute for Health and Care Research. N.O. reports funding support for the present manuscript from UK Research and Innovation; additional funding not related to the present manuscript from Early Detection of Alzheimer's Subtypes; and declares unpaid roles with the DEMON Network (Data Science Lead) and Alzheimer's Association (Steering Committee Member for the ISTAART Artificial Intelligence PIA). J.R. participates on advisory boards for Novartis, Wave Life Sciences, Prevail, Alector, Aviado Bio, and Arkuda Therapeutics; and provides consultancy for Denali, Astex, Takeda, and UCB. A.V. reports funding support for the present manuscript from the National Institute for Health and Care Research. S.C. reports funding support for the present manuscript from the Economic and Social Research Council, National Institute for Health and Care Research, Engineering & Physical Sciences Research Council, and Wellcome Trust. J.W. reports funding support for the present manuscript from the Alzheimer's Society, Royal National Institute for Deaf People, and NIHR UCLH Biomedical Research Centre. All other authors have nothing to declare. Author disclosures are available in the supporting information.

## CONSENT STATEMENT

All survey respondents gave informed consent, in accordance with Declaration of Helsinki guidelines.

## Supporting information

Supporting Information

Supporting Information
