# Supplementary material for: Symptom‐led staging for semantic and non‐fluent/agrammatic variants of primary progressive aphasia
Source: Alzheimers Dement. 2023 Aug 7;20(1):195–210. doi: 10.1002/alz.13415 (PMC10917001; doi:10.1002/alz.13415)
Supplement: Supplementary file 1 — Supporting Information [file ALZ-20-195-s001.docx]

**APPENDICES for “Symptom-led staging for semantic and nonfluent/agrammatic variants of primary progressive aphasia ”, by CJD Hardy et al**

**Appendix Table A1.** Cross-syndromic descriptions of stages presented to caregivers in the consolidation survey

| **PPA stage** | **Daily life impact** |
| --- | --- |
| **1: Very mild** | This stage reflects the earliest clinical manifestations of PPA, but the person may not seek help, attributing problems to stress or ageing. Symptoms may be intermittent, difficult for the person to describe, or recognised only by those who know them well or in hindsight. |
| **2: Mild** | Communication and other less prominent problems with everyday activities are generally evident to others as well as to the person themselves (though sometimes insight may be lacking). |
| **3: Moderate** | The person may now require help managing certain aspects of day-to-day life and will generally have to stop working. Communication difficulties tend to frustrate important goals and social activities. |
| **4: Severe** | The person now requires support with many aspects of daily living and communication is increasingly difficult. They may no longer be able to live independently. |
| **5: Very severe** | Cognitive and behavioural changes are more global in nature, and many are common to all PPA syndromes. Meaningful communication is rarely possible. The person is likely to need help with daily personal care including toileting, and physical symptoms will have developed to the extent that mobility is significantly affected. |
| **6: Profound** | Communication is now no longer possible. The person may lose their ability to respond to their environment and becomes largely immobile |

The Table shows cross-syndromic stage labels that were presented to survey respondents. The stage labels align with those used in the Frontotemporal Dementia Rating Scale^12^. The text in the ‘Daily life impact’ column was developed during exploratory work and adapted from descriptors used in posterior cortical atrophy^36^ and Alzheimer’s disease^11^, and intended to help caregiver survey respondents as a symptom ordering tool. FTD, frontotemporal dementia; PPA, primary progressive aphasia.

**Appendix Table A2.** Summary of survey responses from caregivers for people with svPPA

| **STAGE 1: Very Mild svPPA** | | | | | | | | | | | |
| --- | --- | --- | --- | --- | --- | --- | --- | --- | --- | --- | --- |
| **Original symptom descriptor** | **N responses** | **% present** | **% correct [S1]** | **% S2** | **%S3** | **%S4** | **%S5** | **%S6** | **Action** | **Succinct item wording** | **Neurological interpretation** |
| Problems remembering certain words that are highly specific to the person's interests or hobbies, e.g. a keen gardener might first notice that they are having difficulties with the names for flowers | 27 | 100.0 | **77.8** | 22.2 | 0.0 | 0.0 | 0.0 | 0.0 |  | Difficulty remembering specific familiar (e.g., occupational) vocabulary | Anomia |
| Mood changes, such as becoming more irritable or anxious | 27 | 88.9 | **66.7** | 8.3 | 20.8 | 4.2 | 0.0 | 0.0 |  | Mood changes | Affective alterations |
| Subtle personality changes - e.g. somebody who was previously very careful with money might buy something expensive and out of character | 27 | 63.0 | **58.8** | 17.7 | 17.7 | 5.9 | 0.0 | 0.0 |  | Subtle personality changes | Disinhibition |
| Changes in food preferences or appetite | 25 | 76.0 | **52.6** | 36.8 | 10.5 | 0.0 | 0.0 | 0.0 |  | Changes in appetite | Abnormal eating behaviour |
| Change in libido – an increase or decrease in sexual desire | 25 | 64.0 | **50.0** | 50.0 | 0.0 | 0.0 | 0.0 | 0.0 | Moved to S1 from S2 | Change in libido | Socio-emotional dysfunction |
| Pronunciation errors when reading aloud, especially for unusual 'irregular' words that don't follow regular patterns of pronunciation. For instance, the person may read the word aunt" as "ornt" | 26 | 80.8 | **47.6** | 38.1 | 14.3 | 0.0 | 0.0 | 0.0 |  | Pronunciation errors when reading aloud irregular words (e.g. 'island') | Surface dyslexia |
| Less confidence when engaging in conversations, especially if on the telephone or in groups of people | 27 | 88.9 | **45.8** | 29.2 | 25.0 | 0.0 | 0.0 | 0.0 |  | Less confident engaging in conversations | Impaired discourse |
| Spelling errors, especially for uncommon irregular words that don't follow regular patterns. For example, the person may write the word ache" as "ake" | 27 | 81.5 | **40.9** | 40.9 | 18.2 | 0.0 | 0.0 | 0.0 |  | Spelling errors on less familiar or irregular words (e.g. 'yacht') | Surface dysgraphia |
| **STAGE 2: Mild svPPA** | | | | | | | | | | | |
| **Original symptom descriptor** | **N responses** | **% present** | **%S1** | **% correct [S2]** | **%S3** | **%S4** | **%S5** | **%S6** | **Action** | **Succinct item wording** | **Neurological interpretation** |
| Complicated tasks with multiple steps that the person previously found easy (e.g. cooking an elaborate meal) become more difficult and take the person longer to complete. | 26 | 96.2 | 4.0 | **92.0** | 4.0 | 0.0 | 0.0 | 0.0 |  | Multi-stage tasks more difficult | Executive dysfunction |
| Less self-control, e.g. when it comes to eating chocolate or drinking alcohol. | 26 | 65.4 | 5.9 | **88.2** | 5.9 | 0.0 | 0.0 | 0.0 |  | Gluttonous |  |
| Making lists (‘dictionaries’) of words to be remembered. | 26 | 61.5 | 12.5 | **81.3** | 6.3 | 0.0 | 0.0 | 0.0 |  | Compiles word lists ('dictionaries') | Verbal agnosia |
| Difficulties with reading – stopping reading longer books and instead preferring shorter articles in newspapers or magazines. | 26 | 96.2 | 8.0 | **80.0** | 12.0 | 0.0 | 0.0 | 0.0 |  | Stops reading longer books | Dominant parietal dysfunction |
| Speech is fluent, but seems to ramble and go around the point, e.g. if describing going up on an escalator, they may say, “the moving stairs that take you up”. | 26 | 92.3 | 0.0 | **79.2** | 12.5 | 8.3 | 0.0 | 0.0 |  | Rambling conversation | Circumlocutions |
| Changes in sleeping patterns, e.g. seeming more tired and napping during the day. | 26 | 84.6 | 18.2 | **77.3** | 0.0 | 4.6 | 0.0 | 0.0 |  | Changes in sleeping patterns, e.g. napping | Disordered sleep |
| No longer interested in or enjoys solving crossword puzzles. | 24 | 79.2 | 15.8 | **73.7** | 5.3 | 5.3 | 0.0 | 0.0 |  | No longer enjoys crosswords | Anomia |
| Little insight into the fact that there is anything wrong. | 26 | 73.1 | 26.3 | **73.7** | 0.0 | 0.0 | 0.0 | 0.0 |  | Lacks insight into difficulties | Anosognosia |
| Repetitive/obsessive behaviours | 26 | 92.3 | 8.3 | **66.7** | 20.8 | 4.2 | 0.0 | 0.0 |  | More ‘rigid’ / obsessional | Obsessionality |
| More socially clumsy in situations that require tactfulness. For instance, seeming oddly cheerful when saying hello to somebody at a sombre event like a funeral, or asking a friend or family member a personal question about their appearance. | 26 | 92.3 | 20.8 | **62.5** | 16.7 | 0.0 | 0.0 | 0.0 |  | Socially clumsy and tactless | Socio-emotional dysfunction |
| Particular problems hearing in busy environments, e.g. a noisy room or a dinner party. | 21 | 71.4 | 26.7 | **53.3** | 13.3 | 6.7 | 0.0 | 0.0 |  | Increased hearing difficulty in noise | Central auditory dysfunction |
| †Problems using a computer, e.g being slower to type or enter information | 19 | 84.2 | 12.5 | **43.8** | 43.8 | 0.0 | 0.0 | 0.0 | Added to S2 | Difficulty using computer | Activities of daily living |
| Problems understanding the meaning of more complex or less frequent words | 27 | 85.2 | 26.1 | **43.5** | 30.4 | 0.0 | 0.0 | 0.0 | Moved to S2 from S1 | Difficulty understanding complex or less frequent words | Verbal agnosia |
| †Difficulty assembling new devices/objects | 20 | 60.0 | 16.7 | **33.3** | 33.3 | 16.7 | 0.0 | 0.0 | Added to S2 | Difficulty assembling new devices/objects | Apraxia |
| **STAGE 3: Moderate svPPA** | | | | | | | | | | | |
| **Original symptom descriptor** | **N responses** | **% present** | **%S1** | **% S2** | **% correct [S3]** | **%S4** | **%S5** | **%S6** | **Action** | **Succinct item wording** | **Neurological interpretation** |
| Loss of even common vocabulary, e.g. when asked to get something from the fridge, the person might reply, “What is a fridge?” | 23 | 82.6 | 0.0 | 5.3 | **84.2** | 10.5 | 0.0 | 0.0 |  | Difficulty understanding more common words | Verbal agnosia |
| Some problems with their memory for places or events. | 25 | 100.0 | 8.0 | 12.0 | **80.0** | 0.0 | 0.0 | 0.0 |  | More forgetful | Impaired episodic memory |
| Using very general words like “whatsit” or “that thing” a lot of the time. | 26 | 84.6 | 0.0 | 22.7 | **77.3** | 0.0 | 0.0 | 0.0 |  | Often uses very general words, e.g. "whatsit" | Anomia |
| Marked difficulties in remembering words and names, e.g. forgetting the name of the person who lives down the road. | 25 | 96.0 | 0.0 | 29.2 | **66.7** | 4.2 | 0.0 | 0.0 |  | Difficulty remembering names of people | Anomia |
| Complaining about pains or feelings in the body that don’t seem to have any physical explanation, e.g. headaches, toothache, or pains and feelings in other body parts; and/ or feeling temperature differently to before. | 23 | 69.6 | 0.0 | 31.3 | **62.5** | 6.3 | 0.0 | 0.0 |  | Bodily complaints with no apparent cause | Sensory dysregulation |
| Starting to dislike certain music or other sounds; and/ or complaining of tinnitus or a constant ringing in their ears. | 25 | 52.0 | 23.1 | 0.0 | **61.5** | 15.4 | 0.0 | 0.0 |  | Increased sensitivity to sound / tinnitus | Central auditory dysfunction |
| Finding their way, especially in new places, more difficult. | 24 | 75.0 | 0.0 | 22.2 | **61.1** | 11.1 | 0.0 | 5.6 |  | Difficulty finding way | Topographical agnosia |
| *Getting Yes and No mixed up, for instance saying “No” when asked if they would like a cup of coffee, but really meaning “Yes”. | 20 | 50 | 0.0 | 20.0 | **60.0** | 20.0 | 0.0 | 0.0 | Added to S3 | Confusing 'Yes' and 'No' | Binary reversals |
| Lacking warmth or empathy | 25 | 92.0 | 13.0 | 30.4 | **56.5** | 0.0 | 0.0 | 0.0 |  | Less empathic | Socio-emotional dysfunction |
| Difficulties with questions, e.g. understanding when choices are being given. | 19 | 89.5 | 0.0 | 29.4 | **52.9** | 17.7 | 0.0 | 0.0 |  | Difficulty understanding questions | Receptive agrammatism |
| Showing a sudden love for a particular kind of music or band. | 24 | 33.3 | 0.0 | 37.5 | 62.5 | 0.0 | 0.0 | 0.0 | Removed - not above 50% threshold |  | Central auditory dysfunction |
| **STAGE 4: Severe svPPA** | | | | | | | | | | | |
| **Original symptom descriptor** | **N responses** | **% present** | **%S1** | **%S2** | **%S3** | **% correct [S4]** | **%S5** | **%S6** | **Action** | **Succinct item wording** | **Neurological interpretation** |
| Moving more slowly than before, e.g. when walking from room to room, or getting up from a chair. | 22 | 72.7 | 0.0 | 0.0 | 12.5 | **87.5** | 0.0 | 0.0 |  | Walking more slowly | Parkinsonism |
| Problems recognising household items, e.g. attempting to use bleach as washing-up liquid. | 21 | 90.5 | 0.0 | 5.3 | 10.5 | **84.2** | 0.0 | 0.0 |  | Difficulty recognising household items | Nonverbal agnosia |
| Becoming more withdrawn, not engaging with other people. | 21 | 85.7 | 0.0 | 0.0 | 16.7 | **83.3** | 0.0 | 0.0 |  | Becoming withdrawn / antisocial | Socio-emotional dysfunction |
| Able to carry out some elements of personal care satisfactorily, but needing assistance with some things, e.g. dressing, shaving. | 21 | 85.7 | 0.0 | 5.6 | 11.1 | **72.2** | 11.1 | 0.0 |  | Needs help dressing | Activities of daily living |
| Problems recognising people continue – whilst before the difficulty may have been remembering a person’s name, they may now struggle to recognise that they have even met the person before. | 21 | 100.0 | 0.0 | 0.0 | 28.6 | **66.7** | 4.8 | 0.0 |  | Difficulty recognising familiar people | Prosopagnosia |
| The person makes grammatical as well as spelling errors in emails and notes, etc. | 22 | 86.4 | 0.0 | 5.3 | 31.6 | **63.2** | 0.0 | 0.0 |  | Written grammatical as well as spelling errors | Dysgraphia, expressive agrammatism |
| Problems understanding complicated or long sentences. | 24 | 100.0 | 0.0 | 0.0 | 37.5 | **62.5** | 0.0 | 0.0 |  | Difficulty understanding longer sentences | Receptive agrammatism |
| The person can still speak coherently, but they now rely on ‘stock’ phrases or stories that they will often repeat regardless of context. | 23 | 91.3 | 0.0 | 4.8 | 42.9 | **52.4** | 0.0 | 0.0 |  | Stock phrases | Verbal stereotypies |
| Feeling unsteady and/or falling. | 12 | 66.7 | 0.0 | 0.0 | 12.5 | **37.5** | 37.5 | 12.5 | Moved from S5 to S4 | Poor balance | Postural instability |
| Needing encouraging/ reminding to use the toilet. | 21 | 42.9 | 0.0 | 0.0 | 0.0 | 100.0 | 0.0 | 0.0 | Removed - not above 50% threshold |  | Bladder / bowel dysregulation |
| **STAGE 5: Very Severe svPPA** | | | | | | | | | | | |
| **Original symptom descriptor** | **N responses** | **% present** | **%S1** | **%S2** | **%S3** | **%S4** | **% correct [S5]** | **%S6** | **Action** | **Succinct item wording** | **Neurological interpretation** |
| Difficulties understanding all but the simplest messages. | 10 | 100.0 | 0.0 | 0.0 | 0.0 | 0.0 | **100.0** | 0.0 |  | Difficulty understanding even simple messages | Receptive agrammatism |
| Requiring substantial help with many basic activities, e.g. eating and washing. | 9 | 88.9 | 0.0 | 0.0 | 0.0 | 0.0 | **100.0** | 0.0 |  | Needs help with basic life activities, e.g. eating, washing | Activities of daily living |
| Movements become slower and stiffer | 9 | 66.7 | 0.0 | 0.0 | 0.0 | 0.0 | **83.3** | 16.7 |  | Movements generally stiff and effortful | Parkinsonism |
| Problems recognising themselves in the mirror. | 10 | 60.0 | 0.0 | 0.0 | 16.7 | 0.0 | **83.3** | 0.0 |  | Difficulty recognising self in mirror | Nonverbal agnosia |
| Speech is sparse and largely unintelligible, and now limited to a few words or sounds that may not make sense. | 10 | 50.0 | 0.0 | 0.0 | 20.0 | 0.0 | **80.0** | 0.0 |  | Sparse, largely unintelligible speech | Mutism |
| Increasingly frequent trouble controlling their bladder or bowels. | 10 | 90.0 | 0.0 | 0.0 | 11.1 | 22.2 | **66.7** | 0.0 |  | Urinary / faecal incontinence | Bladder / bowel dysregulation |
| Difficulties recognising food for what it is, e.g. trying to eat inedible objects or eating unheated frozen food without noticing anything is wrong. | 10 | 60.0 | 0.0 | 0.0 | 0.0 | 33.3 | **66.7** | 0.0 |  | Mouthing inedible items | Nonverbal agnosia |
| Able to read and understand only some if any simple words | 13 | 84.6 | 9.1 | 0.0 | 9.1 | 36.4 | **45.5** | 0.0 |  | Difficulty reading simple words | Alexia |
| Problems with swallowing. | 13 | 84.6 | 0.0 | 9.1 | 18.2 | 18.2 | **36.4** | 18.2 |  | Difficulty swallowing | Dysphagia |
| **STAGE 6: Profound svPPA** | | | | | | | | | | | |
| **Original symptom descriptor** | **N responses** | **% present** | **%S1** | **%S2** | **%S3** | **%S4** | **%S5** | **% correct [S6]** | **Action** | **Succinct item wording** | **Neurological interpretation** |
| Unable to perform any acts of daily living and needing to be washed, dressed, fed, etc. by another person. | 8 | 100.0 | 0.0 | 0.0 | 0.0 | 0.0 | 0.0 | **100.0** |  | Needs all basic life activities to be done for them | Activities of daily living |
| There is now almost no speech at all, but the person may make strange sounds (e.g. teeth grinding / popping noises) or laughing, sometimes inappropriately. | 8 | 87.5 | 0.0 | 0.0 | 0.0 | 0.0 | 0.0 | **100.0** |  | Non-verbal sounds in place of speech | Mutism |
| Unable to control most movements without extreme difficulty. The person is mostly confined to chair or bed. | 8 | 50.0 | 0.0 | 0.0 | 0.0 | 0.0 | 0.0 | **100.0** |  | Largely immobile | Parkinsonism |
| The person can no longer write or draw. | 10 | 90.0 | 0.0 | 0.0 | 0.0 | 33.3 | 11.1 | **55.6** |  | Unable to write / draw | Apraxia |
| **Other PPA items** | | | | | | | | | | | |
| **Original symptom descriptor** | **N responses** | **% present** | **%S1** | **%S2** | **%S3** | **%S4** | **%S5** | **%S6** | **Action** | **Succinct item wording** | **Neurological interpretation** |
| Using made-up words that don’t exist, without seeming to be aware that what they have said doesn’t make sense. | 19 | 47.4 | 0.0 | 22.2 | 55.6 | 11.1 | 11.1 | 0.0 | Not added |  | Neologisms |
| Particular problems when speaking in stressful situations, e.g. at a meeting or giving a presentation | 19 | 36.8 | 42.9 | 28.6 | 14.3 | 14.3 | 0.0 | 0.0 | Not added |  | Impaired discourse |
| Problems with ‘spatial orientation’ emerge – the person may have difficulties judging distances or locating objects. | 19 | 21.1 | 25.0 | 0.0 | 75.0 | 0.0 | 0.0 | 0.0 | Not added |  | Visuospatial dysfunction |
| Hallucinations, e.g. seeing or hearing something that isn’t really there. | 19 | 21.1 | 50.0 | 0.0 | 25.0 | 25.0 | 0.0 | 0.0 | Not added |  | Hallucinations |
| **PCA items** | | | | | | | | | | | |
| **Original symptom descriptor** | **N responses** | **% present** | **%S1** | **%S2** | **%S3** | **%S4** | **%S5** | **%S6** | **Action** | **Succinct item wording** | **Neurological interpretation** |
| Difficulty finding things in a handbag, cupboard, etc. | 21 | 47.6 | 0.0 | 30.0 | 50.0 | 20.0 | 0.0 | 0.0 | Not added |  | Visuospatial dysfunction |
| General ‘clumsiness’ (in people who were not previously clumsy), such as knocking things over (inaccurate reaching out), putting a glass down sideways, etc. | 21 | 38.1 | 12.5 | 25.0 | 50.0 | 0.0 | 12.5 | 0.0 | Not added |  | Apraxia, visuospatial dysfunction |
| Experiencing vertigo or other balance problems | 20 | 30.0 | 0.0 | 16.7 | 33.3 | 16.7 | 16.7 | 16.7 | Not added |  | Postural instability |
| Become confused when handling coins due to difficulties telling them apart | 21 | 28.6 | 0.0 | 16.7 | 50.0 | 33.3 | 0.0 | 0.0 | Not added |  | Dominant parietal dysfunction |
| Mixing up left and right | 20 | 15.0 | 0.0 | 33.3 | 33.3 | 33.3 | 0.0 | 0.0 | Not added |  | Dominant parietal dysfunction |
| Little jerky movements in the fingers, arms, or other parts of the body | 21 | 14.3 | 0.0 | 33.3 | 0.0 | 66.7 | 0.0 | 0.0 | Not added |  | Myocolonus |
| Experiencing delusions, e.g. believing that their caregiver is an imposter | 20 | 10.0 | 0.0 | 0.0 | 50.0 | 50.0 | 0.0 | 0.0 | Not added |  | Delusions |
| Difficulty detecting the edge of pavements, paths and steps etc | 21 | 9.5 | 0.0 | 0.0 | 50.0 | 0.0 | 0.0 | 50.0 | Not added |  | Visuospatial dysfunction |
| Experiencing continued deterioration of sensory functions, e.g. partial or complete loss of response to touch | 21 | 9.5 | 0.0 | 0.0 | 0.0 | 50.0 | 0.0 | 50.0 | Not added |  | Sensory dysregulation |
| Difficulty using stairs because of problems with spatial judgment | 20 | 5.0 | 0.0 | 0.0 | 100.0 | 0.0 | 0.0 | 0.0 | Not added |  | Visuospatial dysfunction |
| Be ‘functionally blind’, requiring support in all visually-guided activities | 20 | 5.0 | 0.0 | 0.0 | 100.0 | 0.0 | 0.0 | 0.0 | Not added |  | Visuospatial dysfunction |
| Experiencing odd visual sensations (e.g. occasional changes or washes of colour in the centre or periphery of their vision) | 21 | 4.8 | 0.0 | 0.0 | 0.0 | 100.0 | 0.0 | 0.0 | Not added |  | Early visual dysfunction |
| Being able to see some things but not others, for instance following things that move (e.g. a ball being thrown) but not being able to find static objects (e.g. a ball lying still on the grass in the garden). | 21 | 4.8 | 0.0 | 0.0 | 0.0 | 100.0 | 0.0 | 0.0 | Not added |  | Visuospatial dysfunction |

The table summarises the survey responses given by caregivers for people they cared for with svPPA. If ≥ 50% of respondents indicated a particular symptom was present but of those, a majority indicated that it should have been assigned to an earlier/later stage, that symptom was reassigned accordingly for the final staging (see main text). Original symptom descriptor, the full wording for each symptom that was presented to respondents in the survey. N responses, total number of respondents for that symptom item; %correct, percentage of respondents reporting that symptom was assigned to the correct stage, %Sn, percentage of respondents reporting that symptom was present at a specific stage. Action, summary of decision as to whether to include symptom in final staging system, and where (if cell is blank, this means the item was retained in the stage it was presented to respondents in). Succinct item wording, reduction of original symptom descriptor, homogenised where possible across syndromes. At the end of the survey, respondents were asked additional questions about symptoms present in other PPA syndromes, and in posterior cortical atrophy (PCA): here, respondents had to indicate whether the symptom was present/ absent, and if present, to assign that symptom to a specific stage. *Indicates that an item from another PPA syndrome was endorsed and incorporated into the relevant Stage; †indicates that a PCA symptom was endorsed and incorporated into the relevant Stage. Items that were not endorsed from these additional lists are given at the bottom of the Table.

**Appendix Table A3.** Summary of survey responses from caregivers for people with nfvPPA

| **STAGE 1: Very Mild nfvPPA** | | | | | | | | | | | |
| --- | --- | --- | --- | --- | --- | --- | --- | --- | --- | --- | --- |
| **Original symptom descriptor** | **N responses** | **% present** | **% correct [S1]** | **% S2** | **%S3** | **%S4** | **%S5** | **%S6** | **Action** | **Succinct item wording** | **Neurological interpretation** |
| Particular problems when speaking in stressful situations, e.g. at a meeting or giving a presentation | 44 | 65.9 | **86.2** | 10.3 | 3.4 | 0.0 | 0.0 | 0 |  | Difficulty speaking in stressful (e.g. public) situations | Impaired discourse |
| Repetitive/obsessive behaviours | 42 | 50.0 | **57.1** | 33.3 | 9.5 | 0.0 | 0.0 | 0 |  | More 'rigid' / obsessional | Obsessionality |
| The person makes grammatical as well as spelling errors in emails and notes, etc. | 42 | 76.2 | **56.3** | 28.1 | 12.5 | 3.1 | 0.0 | 0 |  | Written grammatical as well as spelling errors | Dysgraphia, expressive agrammatism |
| †Problems using a computer, e.g being slower to type or enter information | 41 | 78.0 | **38.7** | 16.1 | 38.7 | 0.0 | 0.0 | 6.45 | Added to S1 | Difficulty using computer | Activities of daily living |
| *Change in libido – an increase or decrease in sexual desire | 38 | 55.3 | **38.1** | 19.1 | 28.6 | 9.5 | 0.0 | 4.8 | Added to S1 | Change in libido | Socio-emotional dysfunction |
| **STAGE 2: Very Mild nfvPPA** | | | | | | | | | | | |
| **Original symptom descriptor** | **N responses** | **% present** | **%S1** | **% correct [S2]** | **%S3** | **%S4** | **%S5** | **%S6** | **Action** | **Succinct item wording** | **Neurological interpretation** |
| Complicated tasks with multiple steps that the person previously found easy (e.g. cooking an elaborate meal) become more difficult and take the person longer to complete. | 39 | 92.3 | 8.3 | **69.4** | 19.4 | 2.8 | 0.0 | 0.0 |  | Multi-stage tasks more difficult | Executive dysfunction |
| Getting Yes and No mixed up, for instance saying “No” when asked if they would like a cup of coffee, but really meaning “Yes”. | 41 | 78.0 | 12.5 | **68.8** | 15.6 | 0.0 | 3.1 | 0.0 |  | Confusing 'Yes' and 'No' | Binary reversals |
| Speaking seems to require the person to use more effort than before, taking them longer to say things, and making them more tired after speaking. | 43 | 93.0 | 12.5 | **65.0** | 20.0 | 2.5 | 0.0 | 0.0 |  | Speaking more effortful | Speech apraxia |
| Mood changes, such as becoming more irritable or anxious. | 41 | 90.2 | 18.9 | **62.2** | 18.9 | 0.0 | 0.0 | 0.0 |  | Mood changes | Affective alterations |
| Difficulties finding the right word to say in conversations. | 42 | 95.2 | 20.0 | **60.0** | 15.0 | 5.0 | 0.0 | 0.0 |  | Difficulty finding the right word in conversation | Anomia |
| †Difficulty assembling new devices/objects | 41 | 75.6 | 9.7 | **51.6** | 25.8 | 6.5 | 0.0 | 6.5 | Added to S2 | Difficulty assembling new devices / objects | Apraxia |
| Changes in food preferences or appetite | 41 | 63.4 | 3.9 | **46.2** | 38.5 | 11.5 | 0.0 | 0.0 |  | Changes in appetite | Abnormal eating behaviour |
| Lacking warmth or empathy | 42 | 69.0 | 20.7 | **41.4** | 27.6 | 10.3 | 0.0 | 0.0 |  | Less empathic | Socio-emotional dysfunction |
| Changes in sleeping patterns, e.g. seeming more tired and napping during the day. | 43 | 74.4 | 18.8 | **40.6** | 25.0 | 9.4 | 6.3 | 0.0 |  | Changes in sleeping patterns, e.g. napping | Disordered sleep |
| †Difficulty finding things in a handbag, cupboard, etc. | 42 | 54.8 | 8.7 | **39.1** | 39.1 | 13.0 | 0.0 | 0.0 | Added to S2 | Difficulty finding items in cupboards, etc | Visuospatial dysfunction |
| *Little insight into the fact that there is anything wrong. | 41 | 53.7 | 31.8 | **36.4** | 13.6 | 9.1 | 4.6 | 4.6 | Added to S2 | Lacks insight into difficulties | Anosognosia |
| Particular problems hearing in busy environments, e.g. a noisy room or a dinner party. | 40 | 62.5 | 28.0 | **36.0** | 28.0 | 4.0 | 0.0 | 4.0 |  | Increased hearing difficulty in noise | Central auditory dysfunction |
| **STAGE 3: Moderate nfvPPA** | | | | | | | | | | | |
| **Original symptom descriptor** | **N responses** | **% present** | **%S1** | **%S2** | **% correct [S3]** | **%S4** | **%S5** | **%S6** | **Action** | **Succinct item wording** | **Neurological interpretation** |
| Speech is slow and difficult to understand, and many words are mispronounced. | 38 | 94.7 | 0.0 | 11.1 | **69.4** | 16.7 | 2.8 | 0.0 |  | Mispronouncing words | Speech apraxia |
| Moving more slowly than before, e.g. when walking from room to room, or getting up from a chair. | 41 | 87.8 | 5.6 | 11.1 | **66.7** | 5.6 | 8.3 | 2.8 |  | Walking more slowly | Parkinsonism |
| Less self-control, e.g. when it comes to eating chocolate or drinking alcohol. | 40 | 65.0 | 3.9 | 7.7 | **65.4** | 19.2 | 3.9 | 0.0 |  | Gluttonous | Abnormal eating behaviour |
| Some problems with their memory for places or events. | 38 | 68.4 | 11.5 | 15.4 | **57.7** | 11.5 | 3.9 | 0.0 |  | More forgetful | Episodic memory |
| Difficulties with reading – stopping reading longer books and instead preferring shorter articles in newspapers or magazines. | 42 | 90.5 | 10.5 | 18.4 | **55.3** | 10.5 | 5.3 | 0.0 |  | Stops reading longer books | Dominant parietal dysfunction |
| Difficulties with questions, e.g. understanding when choices are being given. | 42 | 76.2 | 0.0 | 28.1 | **53.1** | 12.5 | 0.0 | 6.3 |  | Difficulty understanding questions | Receptive agrammatism |
| The person can no longer write or draw. | 30 | 86.7 | 0.0 | 0.0 | **46.2** | 19.2 | 23.1 | 11.5 | Moved to S3 from S5 | Unable to write / draw | Apraxia |
| *Problems understanding the meaning of more complex or less frequent words | 41 | 58.5 | 4.2 | 25.0 | **45.8** | 16.7 | 0.0 | 8.3 | Added to S3 | Difficulty understanding complex or less frequent words | Verbal agnosia |
| *Finding their way, especially in new places, more difficult. | 40 | 75.0 | 10.0 | 20.0 | **43.3** | 13.3 | 6.7 | 6.7 | Added to S3 | Difficulty finding way | Topographical agnosia |
| *Problems with ‘spatial orientation’ emerge – the person may have difficulties judging distances or locating objects. | 39 | 53.8 | 4.8 | 33.3 | **42.9** | 4.8 | 14.3 | 0.0 | Added to S3 | Difficulty judging distances, e.g. when driving | Visuospatial dysfunction |
| †Become confused when handling coins due to difficulties telling them apart | 41 | 53.7 | 18.2 | 22.7 | **36.4** | 13.6 | 4.5 | 4.5 | Added to S3 | Difficulty distinguishing coins | Dominant parietal dysfunction |
| More socially clumsy in situations that require tactfulness. For instance, seeming oddly cheerful when saying hello to somebody at a sombre event like a funeral, or asking a friend or family member a personal question about their appearance. | 41 | 53.7 | 4.6 | 31.8 | **36.4** | 27.3 | 0.0 | 0.0 |  | Socially clumsy and tactless | Socio-emotional dysfunction |
| Problems with swallowing. | 36 | 72.2 | 3.9 | 15.4 | **26.9** | 26.9 | 23.1 | 3.9 | Moved to S3 from S4 | Difficulty swallowing | Dysphagia |
| Showing increased preference for things to be done in a certain way/ at a certain time. | 37 | 48.6 | 5.6 | 16.7 | 61.1 | 11.1 | 5.6 | 0.0 | Removed - not above 50% threshold |  | Obsessionality |
| **STAGE 4: Severe nfvPPA** | | | | | | | | | | | |
| **Original symptom descriptor** | **N responses** | **% present** | **%S1** | **%S2** | **%S3** | **% correct [S4]** | **%S5** | **%S6** | **Action** | **Succinct item wording** | **Neurological interpretation** |
| When the person tries to start a conversation, their speech is usually limited to just a few words; if talking to somebody else, the person may ‘latch on’ to a word that person has said and repeat it. | 33 | 97.0 | 3.1 | 0.0 | 15.6 | **68.8** | 6.3 | 6.3 |  | Does not initiate conversation | Adynamia |
| Feeling unsteady and/or falling | 34 | 67.6 | 0.0 | 4.4 | 0.0 | **65.2** | 26.1 | 4.4 |  | Poor balance | Postural instability |
| Movements become slower and stiffer. | 36 | 86.1 | 6.5 | 0.0 | 9.7 | **61.3** | 19.4 | 3.2 |  | Movements generally stiff and effortful | Parkinsonism |
| Able to carry out some elements of personal care satisfactorily, but needing assistance with some things, e.g. dressing, shaving. | 36 | 75.0 | 0.0 | 3.7 | 22.2 | **55.6** | 11.1 | 7.4 |  | Needs help dressing | Activities of daily living |
| Becoming more withdrawn, not engaging with other people. | 36 | 80.6 | 3.5 | 6.9 | 27.6 | **44.8** | 13.8 | 3.5 |  | Becoming withdrawn / antisocial | Socio-emotional dysfunction |
| Able to read and understand only some if any simple words | 25 | 76.0 | 5.3 | 5.3 | 15.8 | **36.8** | 31.6 | 5.3 | Moved to S4 from S5 | Difficulty reading simple words | Alexia |
| Needing reminding/ encouraging to use the toilet. | 33 | 39.4 | 0.0 | 0.0 | 46.2 | 46.2 | 0.0 | 7.7 | Removed - not above 50% threshold |  | Bladder / bowel dysregulation |
| Hallucinations, e.g. seeing or hearing something that isn’t really there. | 34 | 17.6 | 0.0 | 16.7 | 50.0 | 33.3 | 0.0 | 0.0 | Removed - not above 50% threshold |  | Hallucinations |
| **STAGE 5: Very severe nfvPPA** | | | | | | | | | | | |
| **Original symptom descriptor** | **N responses** | **% present** | **%S1** | **%S2** | **%S3** | **%S4** | **% correct [S5]** | **%S6** | **Action** | **Succinct item wording** | **Neurological interpretation** |
| Requiring substantial help with many basic activities, e.g. eating and washing. | 28 | 75.0 | 0.0 | 0.0 | 4.8 | 23.8 | **57.1** | 14.3 |  | Needs help with basic life activities, e.g. eating, washing | Activities of daily living |
| Difficulties understanding all but the simplest messages. | 26 | 76.9 | 0.0 | 5.0 | 5.0 | 35.0 | **50.0** | 5.0 |  | Difficulty understanding even simple messages | Receptive agrammatism |
| Increasingly frequent trouble controlling their bladder or bowels. | 27 | 74.1 | 0.0 | 0.0 | 20.0 | 25.0 | **45.0** | 10.0 |  | Urinary / faecal incontinence | Bladder / bowel dysregulation |
| There is now almost no speech at all, but the person may make strange sounds (e.g. teeth grinding / popping noises) or laughing, sometimes inappropriately. | 26 | 42.3 | 0.0 | 27.3 | 0.0 | 27.3 | 36.4 | 9.1 | Removed - not above 50% threshold |  | Mutism |
| **STAGE 6: Severe nfvPPA** | | | | | | | | | | | |
| **Original symptom descriptor** | **N responses** | **% present** | **%S1** | **%S2** | **%S3** | **%S4** | **%S5** | **% correct [S6]** | **Action** | **Succinct item wording** | **Neurological interpretation** |
| Unable to control most movements without extreme difficulty. The person is mostly confined to chair or bed. | 28 | 75.0 | 4.8 | 0.0 | 0.0 | 0.0 | 33.3 | **61.9** |  | Largely immobile | Parkinsonism |
| Speech is sparse and largely unintelligible, and now limited to a few words or sounds that may not make sense. | 28 | 82.1 | 0.0 | 0.0 | 8.7 | 13.0 | 30.4 | **47.8** |  | Sparse, largely unintelligible speech | Mutism |
| Unable to perform any acts of daily living and needing to be washed, dressed, fed, etc. by another person. | 28 | 82.1 | 0.0 | 0.0 | 0.0 | 26.1 | 30.4 | **43.5** |  | Needs all basic life activities to be done for them | Activities of daily living |
| **Other PPA items** | | | | | | | | | | | |
| **Original symptom descriptor** | **N responses** | **% present** | **%S1** | **%S2** | **%S3** | **%S4** | **%S5** | **%S6** | **Action** | **Succinct item wording** | **Neurological interpretation** |
| Problems recognising household items, e.g. attempting to use bleach as washing-up liquid. | 41 | 43.9 | 0.0 | 27.8 | 38.9 | 27.8 | 0.0 | 5.56 | Not added |  | Nonverbal agnosia |
| Problems recognising people. | 41 | 41.5 | 12.5 | 6.3 | 43.8 | 18.8 | 6.25 | 12.5 | Not added |  | Prosopagnosia |
| Complaining about pains or feelings in the body that don’t seem to have an easy physical explanation, e.g. headaches, toothache, or pains and feelings in other body parts; and/ or feeling temperature differently to before. | 41 | 41.5 | 11.8 | 41.2 | 41.2 | 5.9 | 0.0 | 0.0 | Not added |  | Sensory dysregulation |
| Starting to dislike certain music or sounds; and/ or complaining of tinnitus or a constant ringing in their ears. | 41 | 29.3 | 33.3 | 8.3 | 41.7 | 16.7 | 0.0 | 0.0 | Not added |  | Central auditory dysfunction |
| Showing a sudden love for a particular kind of music or band. | 41 | 14.6 | 0.0 | 16.7 | 66.7 | 0.0 | 0 | 16.67 | Not added |  | Central auditory dysfunction |
| **PCA items** | | | | | | | | | | | |
| **Original symptom descriptor** | **N responses** | **% present** | **%S1** | **%S2** | **%S3** | **%S4** | **%S5** | **%S6** | **Action** | **Succinct item wording** | **Neurological interpretation** |
| Experiencing vertigo or other balance problems | 41 | 48.8 | 25.0 | 15.0 | 40.0 | 15.0 | 0.0 | 5.0 | Not added |  | Postural instability |
| Difficulty detecting the edge of pavements, paths and steps etc | 41 | 46.3 | 0.0 | 21.1 | 36.8 | 15.8 | 26.3 | 0.0 | Not added |  | Visuospatial dysfunction |
| Little jerky movements in the fingers, arms, or other parts of the body | 42 | 42.9 | 22.2 | 11.1 | 33.3 | 22.2 | 11.1 | 0.0 | Not added |  | Myocolonus |
| Mixing up left and right | 41 | 39.0 | 6.3 | 56.3 | 25.0 | 0.0 | 12.5 | 0.0 | Not added |  | Dominant parietal dysfunction |
| Difficulty using stairs because of problems with spatial judgment | 42 | 38.1 | 12.5 | 12.5 | 31.3 | 25.0 | 12.5 | 6.3 | Not added |  | Visuospatial dysfunction |
| General ‘clumsiness’ (in people who were not previously clumsy), such as knocking things over (inaccurate reaching out), putting a glass down sideways, etc. | 42 | 35.7 | 26.7 | 20.0 | 40.0 | 13.3 | 0.0 | 0.0 | Not added |  | Apraxia, visuospatial dysfunction |
| Experiencing continued deterioration of sensory functions, e.g. partial or complete loss of response to touch | 39 | 28.2 | 9.1 | 18.2 | 18.2 | 36.4 | 0.0 | 18.2 | Not added |  | Sensory dysregulation |
| Being able to see some things but not others, for instance following things that move (e.g. a ball being thrown) but not being able to find static objects (e.g. a ball lying still on the grass in the garden). | 41 | 22.0 | 44.4 | 11.1 | 11.1 | 22.2 | 11.1 | 0.0 | Not added |  | Visuospatial dysfunction |
| Be ‘functionally blind’, requiring support in all visually-guided activities | 41 | 12.2 | 20.0 | 20.0 | 20.0 | 40.0 | 0.0 | 0.0 | Not added |  | Visuospatial dysfunction |
| Experiencing delusions, e.g. believing that their caregiver is an imposter | 42 | 11.9 | 20.0 | 40.0 | 0.0 | 40.0 | 0.0 | 0.0 | Not added |  | Delusions |
| Experiencing odd visual sensations (e.g. occasional changes or washes of colour in the centre or periphery of their vision) | 42 | 7.1 | 33.3 | 0.0 | 66.7 | 0.0 | 0.0 | 0.0 | Not added |  | Early visual dysfunction |

The table summarises the survey responses given by caregivers for people they cared for with nfvPPA. If ≥ 50% of respondents indicated a particular symptom was present but of those, a majority indicated that it should have been assigned to an earlier/later stage, that symptom was reassigned accordingly for the final staging (see main text). Original symptom descriptor, the full wording for each symptom that was presented to respondents in the survey. N responses, total number of respondents for that symptom item; % present, percentage of respondents reporting that symptom as present in the person they care(d) for, at any stage of the disease; % earlier, percentage of respondents reporting that symptom occurred at an earlier stage than assigned; % correct, percentage of respondents reporting that symptom was assigned to the correct stage; % later, percentage of respondents reporting that symptom occurred at a later stage. Action, summary of decision as to whether to include symptom in final staging system, and where (if cell is blank, this means the item was retained in the stage it was presented to respondents in). Succinct item wording, reduction of original symptom descriptor, homogenised where possible across syndromes. At the end of the survey, respondents were asked additional questions about symptoms present in other PPA syndromes, and in posterior cortical atrophy (PCA): here, respondents had to indicate whether the symptom was present/ absent, and if present, to assign that symptom to a specific stage. *Indicates that an item from another PPA syndrome was endorsed and incorporated into the relevant Stage; †indicates that a PCA symptom was endorsed and incorporated into the relevant Stage.

**Appendix Table A4.** Prototipo de escala de deterioro funcional para la afasia progresiva primaria, la *Ayuda a la Planificación de la Progresión de la APP (PPA-Squared)*

| **APP-vs** | | | | | |
| --- | --- | --- | --- | --- | --- |
| **Dominio** | **Nivel 0**  **Presintomático** | **Nivel 1**  **Muy leve** | **Nivel 2**  **Leve** | **Nivel 3**  **Moderado** | **Nivel 4**  **Severo** |
| Comunicación | Sin cambios | Es posible que no entienda vocabulario específico (p.ej., ocupacional)  Errores de ortografía con verbos irregulares | No entiende palabras poco comunes | No entiende palabras poco comunes | Habla estereotipada, volviéndose ininteligible |
| Pensamiento no verbal y personalidad | Sin cambios | Cambios en el estado de ánimo, el apetito, la libido | Las tareas con varios pasos son más difíciles  Tiene comportamientos socialmente inapropiados  No se da cuenta de la enfermedad  Aumento de la dificultad auditiva en ambientes ruidosos | Más olvidadizo.  Dificultades de navegación | Dificultades para reconocer personas familiares y artículos del hogar  Introversión social |
| Cuidado personal y bienestar | Sin cambios | Sin cambios | Cambios en el patrón de sueño  Glotonería | Quejas corporales sin explicación aparente  Sensibilidad al ruido/tinnitus | Requiere ayuda para vestirse y otras actividades básicas de la vida diaria  Incontinencia  Dificultad para tragar  Dificultad para caminar, equilibrio afectado. |
| **APP-nf** | | | | | |
| **Dominio** | **Nivel 0 Presimtomático** | **Nivel 1**  **Muy leve** | **Nivel 2**  **Leve** | **Nivel 3**  **Moderado** | **Nivel 4**  **Severo** |
| Comunicación | Sin cambios | Errores gramaticales y ortográficos  Dificultades para hablar en situaciones estresantes (p.ej., en público) | Dificultad para encontrar las palabras en conversaciones  Habla con más esfuerzo  Confunde “sí” y “no” | Comete errores en la pronunciación de palabras  Ya no puede escribir/dibujar  Puede tener dificultad para comprender preguntas y palabras poco frecuentes | Habla escasa, a veces ininteligible  No comprende mensajes sencillos |
| Pensamiento no verbal y personalidad | Sin cambios | Cambios en la libido  Mas rígido, obsesivo | Las tareas con varios pasos son más difíciles  Cambios de humor y apetito  Muestra menos empatía  No se da cuenta de la enfermedad  Aumento de la dificultad auditiva en ambientes ruidosos | Dificultades de navegación  Más olvidadizo  Habilidades visuoespaciales afectadas  Tiene comportamientos socialmente inapropiados | Introversión social |
| Cuidado personal y bienestar | Sin cambios | Sin cambios | Cambios en los patrones del sueño | Camina más lento  Dificultades para tragar  Glotonería | Movimiento con rigidez y esfuerzo Requiere ayuda para las actividades básicas de la vida diaria  Incontinencia  Dificultades de equilibrio |

Esta tabla resume los síntomas clave para el funcionamiento de la vida diaria que surgen durante el curso clínico de la variante semántica (fila superior) y la variante no fluente/agramatical de la afasia progresiva primaria (fila inferior), como se identificó en las encuestas a los cuidadores. Los síntomas están clasificados en tres dominios funcionales utilizados al recopilar datos de los cuidadores (consulte el texto, Figuras 2 y 3). Los síntomas dentro de cada celda están enumerados en orden de frecuencia (los más frecuentes en la parte superior), de acuerdo con las respuestas recibidas en las encuestas de cuidadores (consulte las Figuras 2 y 3). Esta escala está diseñada para ser utilizada como una medida de la progresión y/o gravedad de la enfermedad: un médico administraría la escala al paciente y al cuidador como parte de la entrevista clínica; el nivel de deterioro se valora para cada dominio por separado y al final se combinan para generar una puntuación total. Por lo tanto, si bien existe una correspondencia general entre los 'niveles' crecientes aquí y las 'etapas' presentadas en las Figuras 2 y 3, un paciente individual podría mostrar (por ejemplo) síntomas de comunicación de 'nivel 2' pero síntomas de pensamiento no verbal y personalidad de 'nivel 1', etc (ver texto). Tenga en cuenta que la escala es provisional a la espera de una mayor validación (ver texto).
